# Dimerizing Heptamethine Cyanine Fluorophores from the Meso Position: Synthesis, Optical Properties, and Metal Sensing Studies

**DOI:** 10.1021/acs.orglett.5c01620

**Published:** 2025-06-09

**Authors:** Tarek Erfan Ahmed, Maged Henary

**Affiliations:** † Department of Chemistry, 1373 Georgia State University, Atlanta, Georgia 30303, United States; ‡ Center For Diagnostics and Therapeutics, Georgia State University, Atlanta, Georgia 30303, United States

## Abstract

A series of eight dimeric heptamethine cyanine fluorophores were synthesized, purified, and produced in moderate yields by connecting two heptamethine dyes from their meso position. Their physicochemical properties were predicted, and their optical properties and photothermal stability were studied. They showed selective absorbance and fluorescence quenching in the presence of copper­(II) ions. This study serves as a basis for exploring the effects of dimerization of heptamethine cyanine fluorophores and their potential use for various biomedical applications.

## Graphical abstract

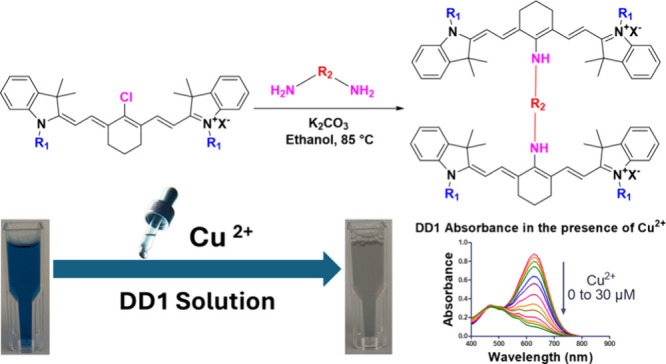

Cyanine fluorophores, characterized by their unique structural features and optical properties, have emerged as indispensable tools in various biomedical applications.^1–10^ Their ability to absorb and emit light in the near-infrared (NIR) region of the electromagnetic spectrum offers distinct advantages, including deeper tissue penetration, reduced autofluorescence, and enhanced signal-to-noise ratios.^3,4^ Interest in dimerizing cyanine dyes has developed over the years to achieve new applications that were nonachievable with their monomeric counterparts.^11–14^ Initial studies for dimeric cyanine fluorophores linked the two subunits from the nitrogen atoms in the heterocyclic ring by different flexible linkers.^13–16^ The produced dimeric dyes had high hydrophobicity, which resulted in their aggregation when placed in aqueous solution and the formation of a clamshell-like conformation that led to quenching of fluorescence.^13,14,16^ However, when these fluorophores bound to a biomolecule like albumin, this clamshell conformation was dissociated, and the fluorescence was regained with higher quantum yield.^13,16^ Some studies used the hydrophobic properties of these dyes to bind to the fatty components in fingerprints to allow their latent visualization in crime scenes. This interaction gave these fluorophores a unique forensic application.^11,17^

Other applications include binding to metals, as the dimeric dyes have stronger complexing ability to metals compared to the monomeric dyes, as they have more electronegative atoms that can bind to metals.^18,19^ Complexation to metals caused changes in the optical properties of these dyes, which allowed their use in metal sensing applications.^20,21^

The human body needs copper­(II) for many biological functions, as it is an essential micronutrient. But, either too high or too low levels of it can seriously harm human health.^22–24^ That is why it is so important to have efficient methods for detecting and measuring copper­(II) ions accurately. Interest in developing dye-based chemosensors for copper­(II) detection has risen in recent years.^25–28^ Not only did they show high selectivity and sensitivity, but also they had the potential for visual detection.^25,26^ The concept behind these chemosensors is the change in the optical properties upon binding to copper­(II) ions.^29–32^ This change can be anything from a shift in the wavelength of absorption or emission,^33^ a change in the fluorescence intensity, or a colorimetric response.^29,31,32^ The mechanism behind this is usually a specific interaction between the copper ion and the dye such as coordination of copper with Lewis base sites in the dye molecule.

Fluorescence quenching has been a method of copper­(II) ion detection for many small-molecule probes including anthracene,^29,34^ BODIPY,^35^ dansyl,^36^ fluorescein,^37^ pyrene,^38^ quinoline,^39^ and quinazoline^40^-based probes. It was also used for merocyanine-based probes, which had a wavelength of ∼500 nm.^41^ To the best of our knowledge, dimeric heptamethine cyanine fluorophores have never been used for detection of copper­(II) ions, and thus the novelty of our work.

Herein, a different type of heptamethine cyanine fluorophore dimer was developed and studied, focusing on its potential use for detecting metals in biomedical applications. The method used incorporated the connection of two heptamethine cyanine molecules from their central meso positions using two amine-functionalized linkers. This dimerization concept was reported earlier by Mojzych et al.,^42^ but our recent work has several advantages over their study. First, the synthetic method reported in our work included using ethanol in the dimerization step, which caused the yields to be much higher than their reported yields (26–49% vs 10–15%). Another advantage is the full characterization using ^1^HNMR, ^13^CNMR, and HRMS, spectral data of our new dye derivatives, the extensive photophysical studies, and the reported sensing data. The previous work only included characterization using HRMS and ^1^H NMR without providing any Supporting Information and determining absorbance wavelength maxima only in methanol. Our new dimers were synthesized with high purity using different column chromatography and recrystallization techniques, and then their optical properties were studied. The studies included measuring the absorbance, fluorescence intensity, quantum yield, and molecular brightness. The physicochemical characteristics were predicted with Chemaxon MarvinSketch software,^43^ and their photothermal stability was tested. Their capacity for metal sensing was evaluated by observing absorbance and fluorescence intensity shifts in the presence of increasing levels of different metal ions. This work examined how linking heptamethine cyanine fluorophores centrally affected their fundamental physicochemical and optical properties. Using varied linkers helped to clarify how the molecular structure influenced photophysical behavior and assessed the suitability of these dimers for biomedical roles such as fluorescence imaging and sensor development.

## Results and Discussion

A series of dimeric heptamethine cyanine dyes were synthesized, as shown in Schemes 1–3, starting from phenylhydrazine and cyclohexanone. First, phenylhydrazine **1** reacted with 3-methyl-2-butanone **2** via the Fischer indole synthesis to form the trimethyl-substituted indole **3**, which was alkylated with different alkyl halides to produce the indolium salts **4**. The dialdehyde linker **6** was synthesized through the Vilsmeier–Haack chloroformylation from cyclohexanone **5**, dimethylformamide (DMF), and phosphorus oxychloride (POCl_3_) in dichloromethane (DCM). The indolium salts **4** were reacted with dialdehyde linker **6** to form heptamethine cyanine dyes **7**. After the formation of the heptamethine dyes, they were allowed to react with various linkers that connected two molecules of the heptamethine dyes through their central meso position by substitution of the chlorine atom with a nitrogen atom in the linker through a radical-nucleophilic aromatic substitution (S_RN_1) reaction to form the dimeric dyes **DD1–8**.

**Scheme 1:**
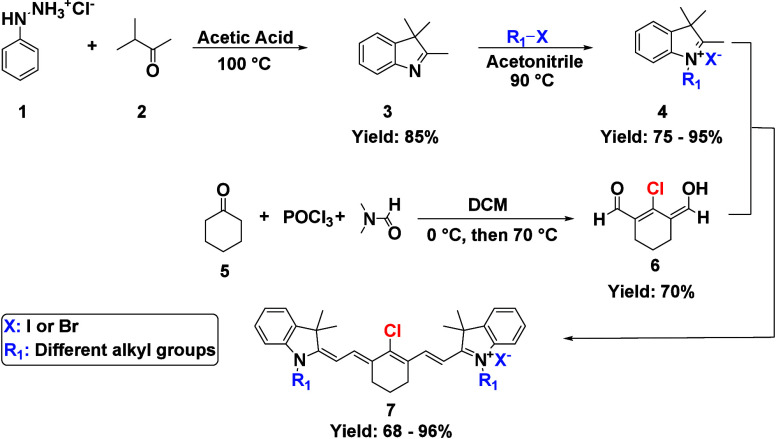
Synthesis of the Heptamethine Monomeric Dyes

**Scheme 2:**
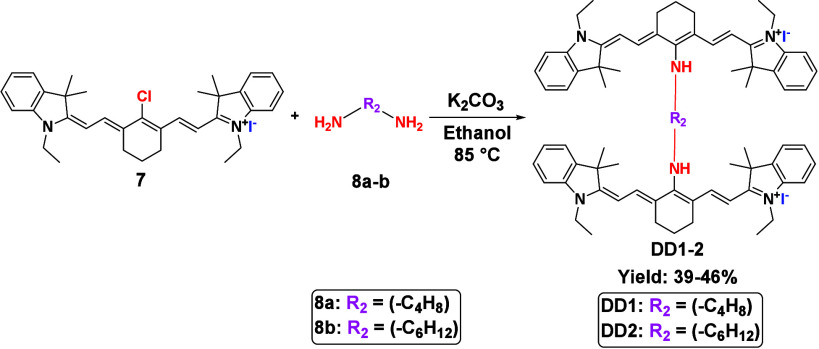
Synthesis of the Ethyl-Substituted Indolium Dimeric Cyanine Dyes (**DD1** and **DD2**)

**Scheme 3:**
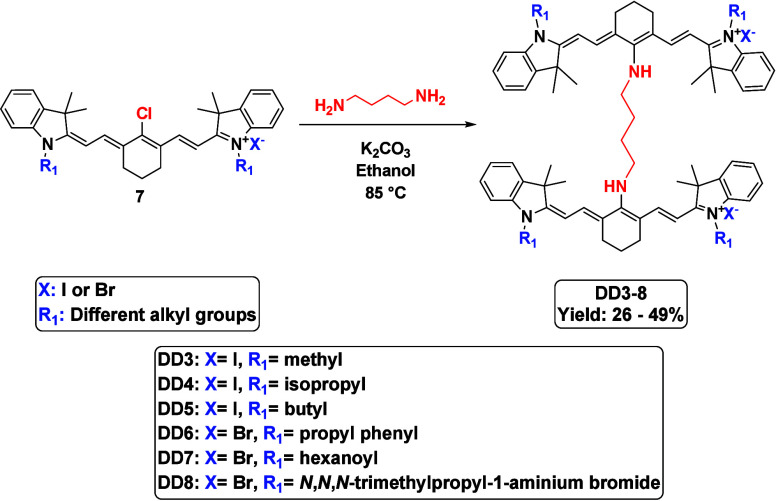
Synthesis and Structures of the Synthesized Dimeric Cyanine Dyes with 1,4-Diaminobutane (**DD3**–**8**)

To investigate the influence of linker structure on the properties of the dimeric heptamethine cyanine dyes, two dimers were synthesized first. Each dimer incorporated a distinct linker moiety, differing in its alkyl chain length. The resulting dimeric dyes **DD1** and **DD2** facilitated a comparative analysis of the impact of linker length on the overall dye properties. Scheme 2 shows the synthesis of the two dimeric dyes synthesized using the ethyl-substituted indolium salt.

The synthesized dimeric dyes underwent computational prediction of their physicochemical properties. Subsequently, experimental investigations focused on characterizing their optical properties, photostability, and metal-sensing capabilities via absorbance and fluorescence spectroscopy. **DD1**, incorporating a 1,4-diaminobutane linker, exhibited superior optical performance, prompting the synthesis of a focused series of derivatives as shown in Scheme 3.

Substituent modifications on the indolium salts were implemented to systematically probe their influence on both physicochemical properties (e.g., distribution coefficient and polarizability) and optical characteristics. Specifically, six more derivatives of the 1,4-diaminobutane-linked dimers were synthesized (Scheme 3), given their excellent optical properties and metal-sensing potential. This structure–property investigation aimed to elucidate the effects of the substituent functionality on the overall dye performance.

### Physicochemical Properties

Chemaxon Marvinsketch software was used to predict the physicochemical properties of the synthesized dimeric dyes, and they are shown in Table S2.^43^ The predicted properties included the distribution coefficient (Log *D*), which is indicative of the hydrophobicity of the dye^2,44^ and changes widely with varying the linker length and the substituents on the indolium ring. More polar substituents such as the quaternary ammonium group drastically decreased the Log *D* of fluorophore **DD8**. In contrast, highly hydrophobic fluorophores such as **DD4–6** exhibited high Log *D*. The number of rotatable bonds was also calculated, which is important for the flexibility of the molecule and the effectiveness of the charge transfer.^45,46^ **DD1**, **DD3**, and **DD4** had the lowest number of rotatable bonds and so were expected to have the most effective charge transfer and hence higher extinction coefficient.^45,47^

Some geometrical descriptors were also calculated as the surface area, total polar surface area, and the volume of the molecule, which are all important descriptors for the substance that are useful especially when studying biological applications of the fluorophores.^46,48^ The dimeric dyes with large substituents such as **DD6–8** exhibited the largest values of these descriptors. The number of hydrogen bond donors (HBD) and acceptors (HBA) were also calculated, because hydrogen bonds are important for the interactions of the fluorophores intermolecularly and with biomolecules.^49,50^ The dimer with a hexanoate substituent, **DD7**, had the highest values of HBD and HBA due to the presence of extra oxygen atoms. Polarizability describes how easily a molecule’s electron cloud can be distorted by an external electric field.^51^ For fluorophores, this property is crucial because it dictates how they interact with their environment, influencing their spectral properties like absorption and emission wavelengths.^51^ A similar trend to the number of HBA was observed with the polarizability, where the oxygen atoms have higher ability to be polarized, increasing its calculated value.

### Optical Properties

The optical properties of the dimeric dyes were measured in various solvents with different characteristics. The solvents used were ethanol, a polar protic organic solvent, DMSO, a polar aprotic organic solvent, 4-(2-hydroxyethyl)­piperazine-1-ethane-sulfonic acid (HEPES) buffer, an aqueous-based buffer solution, and phosphate-buffered saline (PBS), another aqueous-based buffer solution used to mimic the body’s aqueous environment. The dyes’ optical properties differ in those various solvents due to the change in the medium and the solvatochromic effect because of the change in the solvent dielectric constant and polarity level.^52^ The FDA-approved fluorophore indocyanine green (ICG) was used as a reference and was used as a standard in the calculation of the quantum yield of fluorescence (Φ_f_).^53,54^ Tables 1 andS3–S6 show the optical properties in the different solvents.

**Table 1:** Dimeric Dye Optical Properties in HEPES Buffer

Dye	Absorbance Wavelength Maxima (nm)	Excitation Wavelength (nm)	Emission Wavelength (nm)	Stokes Shift (nm)	Extinction coefficient (M^–1^cm^–1^)	Quantum Yield of Fluorescence (%)	Molecular Brightness (M^–1^ cm^–1^)
DD1	615	600	803	188	87,100	1.6	1,378
DD2	615	600	770	155	67,800	1.5	1,029
DD3	625	600	760	135	101,600	5.2	5,326
DD4	620	600	766	146	90,900	2.1	1,940
DD5	605	600	750	145	52,600	3.2	1,674
DD6	620	600	779	159	57,500	1.6	910
DD7	625	600	758	133	16,400	11.4	1,863
DD8	605	600	752	147	74,100	7.1	5,288
ICG^65^	778	720	802	24	148,000	2.9 ^65^	4,292

The synthesized heptamethine dimeric dyes exhibited absorbance maxima in the range of 605 to 640 nm due to the perturbation of the π-electron system, which led to increasing the energy gap between the ground and excited state and so blue shifting the wavelength compared to their original monomeric heptamethine dyes. The blue shift may also result from an intramolecular charge transfer (ICT) or excited-state proton transfer;^55^ another reason that was suggested is the symmetry breaking caused by the amino alkyl meso substitution.^56,57^

The dimeric dyes were excited at 600 nm to get their emission spectra; 600 nm was used as the excitation wavelength because it was the closest wavelength to their absorption maxima wavelength that did not cause overlap between the excitation and emission peaks. The emission wavelength for the dimeric dyes lay in the 740 to 803 nm region. The blue shift observed in absorbance was not observed in the fluorescence because the relaxation pathways were different, where the molecule relaxed using some nonradiative pathway or vibrational relaxation to a lower excited state before it relaxed by fluorescence to the ground state. This combined relaxation pathway decreased the energy gap and increased the wavelength of emission. Hence this variation in the effect of the nitrogen caused a large Stokes shift of 105 to 188 nm, which is very beneficial in decreasing the background interference from the excitation source and helps in increasing the image quality in bioimaging applications.^58,59^

The extinction coefficient values of the dimeric dyes ranged from 16,400 to 130,100 M^–1^ cm^–1^. They were the highest in ethanol and DMSO due to enhanced oscillator strength because of the favorable solvent–dye interactions and the reduced aggregation due to the dyes’ better solubility in organic solvents.^60^ Previous reports showed that cyanine dyes tend to aggregate in water and water-based buffers;^61,62^ that is why it was proposed that the synthesized dimeric dyes had lower solubility in buffer solutions, and this was evidenced by their broad absorption spectra. The quantum yield of fluorescence showed a similar trend in the effects of the solvent, where it was largest in ethanol and DMSO for most of the dyes due to the better solubility and decreased aggregation. It ranged from 11.6% to 43.9% in ethanol and DMSO and from 1.1% to 11.4% in HEPES and PBS buffers.

Molecular brightness is the product of the molar extinction coefficient and the quantum yield of fluorescence, and hence, its values will be dependent on both properties. That is why its values had a similar trend of being higher in organic solvents and lower in aqueous buffers. The values ranged from 4,275 to 48,569 M^–1^ cm^–1^ in ethanol and DMSO and from 514 to 5,326 M^–1^ cm^–1^ in HEPES and PBS buffers. Interestingly, the molecular brightness of many of the dimeric dyes was higher than that of the FDA-approved fluorophore ICG, due to their higher values of extinction coefficient and quantum yield of fluorescence. This is a great advantage for these dimeric dyes, as this makes them promising candidates for imaging applications due to higher signal to noise ratio, increased sensitivity, reduced photobleaching, and better resolution.^63,64^

The effect of changing the substituents on the indolium ring was subtle. Only dimeric dyes with hexanoic acid-substituted indolium **DD7** showed lower molar extinction coefficients. This could be attributed to its higher ability to form hydrogen bonds, leading to more intermolecular interactions and hence higher aggregation, which reduced the extinction coefficient due to changes in the electronic environment and energy transfer processes within the aggregates. Finally, it was also more likely to interact with polar solvents, which could disrupt its planarity and decrease the conjugation.

### Photothermal Stability Study

The photothermal stability of the synthesized dimeric dyes was studied by comparing the absorbance of the fluorophores over time for 72 h under two conditions and compared to ICG. One set of fluorophores was put in the dark, covered by a foil plate, and the other set was continuously irradiated by a 6000 mW 254 nm UV lamp light placed 10 cm away from the dye’s solutions. The measured absorbances over time are shown in Figures 1 and S33. Under dark conditions, most of the synthesized dimeric dyes were stable and their absorbance almost did not change except **DD3**, which decreased by 17% over the course of 72 h. Under light irradiation, many of the dimeric dyes showed good photostability especially for the first 24 h. At 48 h, six of the dimeric dyes preserved their absorbance more than ICG, where only **DD2** and **DD3** were less stable than ICG. At 72 h, most of the dimeric dyes and ICG lost more than 50% of their absorbance. Only fluorophores **DD4**, **DD6**, and **DD8** preserved more than 50% of their absorbance, and **DD8** was the most stable, keeping 68% of its absorbance after 72 h.

**Figure 1:**
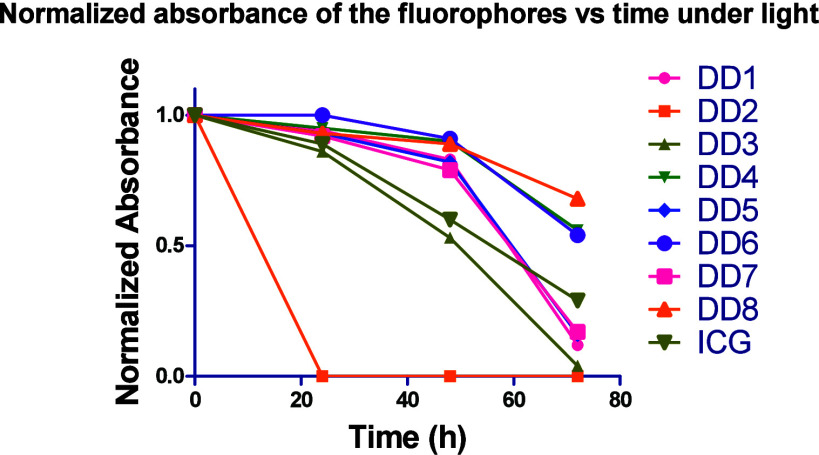
Photothermal stability of dimeric dyes **DD1–8**. Their normalized absorbance over time under irradiation with a 6000 mW 254 nm UV lamp placed 10 cm from the dye solution (6 μM in ethanol) at 25 °C.

### Metal Sensing Study

Fluorophore **DD1** as the lead dimeric dye was tested for metal sensing, and it showed sensitivity toward Cu^2+^ ions selectively. Its absorbance was measured at increasing concentrations of different metal ions, and it selectively showed a hypochromic effect with increasing concentrations of Cu^2+^ ions (Figure 2). It did not show the same effect with other metal ions tested. The absorbance was measured in the presence of other metal ions including Ag^+^, Li^+^, Na^+^, K^+^, Cr^3+^, Ni^2+^, Co^2+^, Zn^2+^, Hg^2+^, and Ca^2+^, and it did not show any response toward them, which makes it selective for Cu^2+^ sensing (Figures 3 and S34).

**Figure 2:**
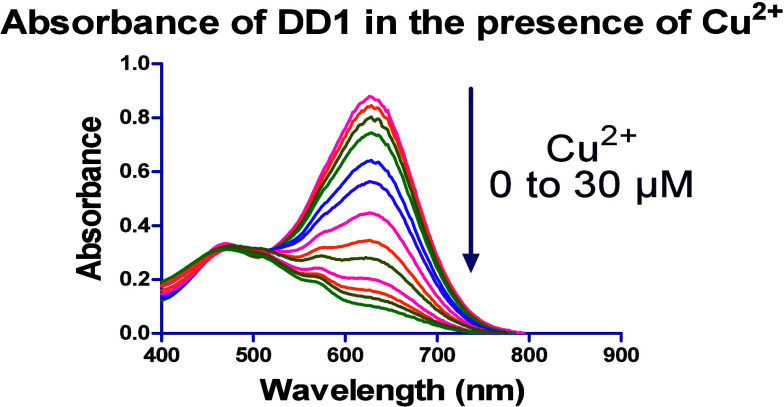
Effect of Cu^2+^ on the absorbance of **DD1** in 50 mM HEPES buffer.

**Figure 3:**
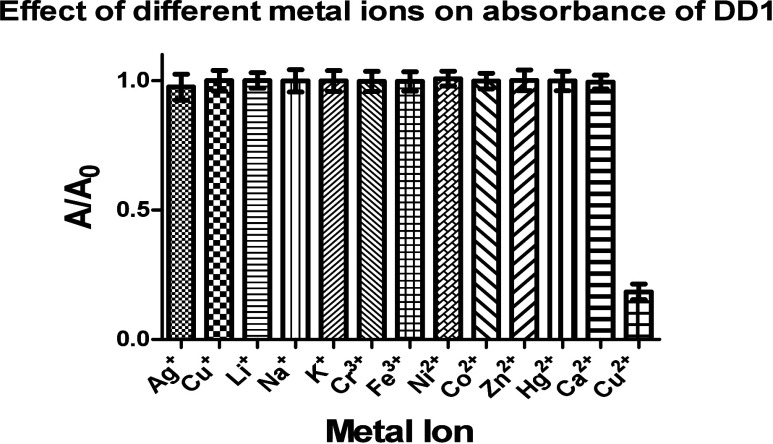
Effect of different metal ions on the absorbance of **DD1** in 50 mM HEPES buffer.

The fluorescence intensity of **DD1** was also measured at increasing concentrations of these metal ions, and a similar effect was observed with Cu^2+^, where it showed a decrease in fluorescence intensity (fluorescence quenching) with the increment of Cu^2+^ and not with the other metal ions (Figures S35–S37). The limit of detection (LOD) and limit of quantitation (LOQ) were calculated by using a calibration curve plotting the change in absorbance or fluorescence against Cu^2+^ concentration. From the absorbance calibration curve, the LOD was calculated to be 2.5 μM and the LOQ was calculated to be 7.6 μM, and from the fluorescence calibration curve, the LOD was 2.2 μM and the LOQ was 6.7 μM, which are comparable to the values from the absorbance curve (Figures S38 and S39). The same study was performed on **DD2**, and it gave comparable results (Figures S40–S47). This metal sensing with Cu^2+^ will be studied in more detail in future work, where the mechanism of interaction, the stoichiometry of the complex, and the binding constant will be studied experimentally and computationally to determine them.

## Conclusion

In summary, eight dimeric heptamethine cyanine fluorophores were synthesized by connecting two heptamethine cyanine molecules from their central meso position. They showed good molar extinction coefficients, quantum yields of fluorescence, and high molecular brightness compared to ICG. They showed good photostability for 48 h, and three of them were more stable than ICG. For metal sensing, the dimeric dyes showed colorimetric hypochromic behavior of absorbance and fluorescence quenching upon increasing Cu^2+^ ion concentration selectively. The metal sensing studies need more work to be conducted, which is the future direction for our study. This work presents a different approach for dimerizing cyanine fluorophores from the central meso position and paves the way for exploring more derivatives that have variable physicochemical and/or optical properties and testing their potential for different biomedical applications.

## Supplementary Material



## Data Availability

The data underlying this study are available in the published article and its Supporting Information.

## Notes

Experimental procedure, ^1^H NMR and ^13^C NMR of all new compounds, tables of predicted physicochemical properties, optical properties in different solvents, absorbance and fluorescence spectra, photostability, and metal sensing graphs (PDF)
