# Supplementary material for: Dimerizing Heptamethine Cyanine Fluorophores from the Meso Position: Synthesis, Optical Properties, and Metal Sensing Studies
Source: Org Lett. 2025 Jun 9;27(25):6623–9. doi: 10.1021/acs.orglett.5c01620 (PMC12210257; doi:10.1021/acs.orglett.5c01620)
Supplement: Supplementary file 1 [file ol5c01620_si_001.pdf]

Supporting Information

Dimerizing heptamethine cyanine fluorophores from the meso position: Synthesis, optical properties and metal sensing studies

Tarek Erfan Ahmed[1], Maged Henary*[1,2]
[1]Department of Chemistry, Georgia State University, Atlanta, Georgia, 30303
[2]Center For Diagnostics and Therapeutics, Georgia State University, Atlanta, Georgia, 30303

*Address correspondence to
Dr. Maged Henary
Professor and Associate Chair of Chemistry
Department of Chemistry
Georgia State University
Atlanta, Georgia 30303
USA
mhenary1@gsu.edu
Phone: 404-413-5566

Table of Contents

1. List of abbreviations 3
2. Experimental Section 3
   2.1. Materials and Methods 3
   2.2. Chemistry 4
      2.2.1. General synthetic procedure of the indolium salts (4) 4
      2.2.2. Synthesis of Vilsmeier–Haack linker of the heptamethine cyanine dyes (6) 4
      2.2.3. General synthetic procedure for the heptamethine cyanine dyes (7) 4
      2.2.4. General synthetic procedure for the dimeric cyanine dyes (DD1-DD8) 4
   2.3. Optical Studies 7
      2.3.1. Quantum yield of fluorescence (Φ_f) calculation 7
   2.4. Photothermal stability Studies 7
   2.5. Metal Sensing Studies 7
3. ^{1}H NMR, ^{13}C NMR, and HRMS Spectra of the synthesized dimeric dyes 9
4. Physicochemical Properties of the synthesized dimeric dyes 33
5. Tables of optical properties in different solvents 33
6. Absorbance and Emission Spectra of the synthesized dimeric dyes 35
7. Photothermal stability Studies 43
8. Metal Sensing Studies 44
   8.1. Effect of increasing concentrations of metal ions on DD1 Absorbance 44
   8.2. Effect of increasing concentrations of metal ions on DD1 Fluorescence 45
   8.3. Effect of Cu^{2+} and other metal ions on the fluorescence intensity of DD1 46
   8.4. Change in DD1 absorbance with increasing concentrations of copper (II) ions 47
   8.5. Change in DD1 fl intensity with increasing concentrations of copper (II) ions 47
   8.6. Effect of increasing concentrations of metal ions on DD2 Absorbance 48
   8.7. Effect of increasing concentrations of metal ions on DD2 Fluorescence 49
   8.8. Effect of Cu^{2+} and other metal ions on the absorbance of DD2 50
   8.9. Effect of Cu^{2+} and other metal ions on the fluorescence intensity of DD2 51
   8.10. Change in DD2 absorbance with increasing concentrations of copper (II) ions 52
   8.11. Change in DD2 fl intensity with increasing concentrations of copper (II) ions 52
9. References 52

1. List of abbreviations

- TLC: Thin layer chromatography
- DCM: Dichloromethane
- DMF: Dimethylformamide
- EtOH: Ethanol
- DMSO: Dimethyl sulfoxide
- HEPES: 4-(2-hydroxyethyl)-1-piperazineethanesulfonic acid
- PBS: Phosphate-buffered saline
- Abs: Absorbance
- Flu: Fluorescence
- LOD: Limit of Detection
- LOQ: Limit of Quantitation

2. Experimental Section

2.1. Materials and Methods

Chemicals used in the synthesis are American Chemical Society or HPLC grade, purchased from Sigma Aldrich (Saint Louis, MO), Thermo Fisher Scientific and TCI America (Waltham, MA). The ^{1}H-NMR (400 MHz) and ^{13}C-NMR (100 MHz) spectra were recorded using a Bruker Avance spectrometer with DMSO-d_6 (Cambridge Isotope Laboratories, Andover, MA), $CDCl_3$ (Sigma-Aldrich, Burlington, MA), and MeOD (Sigma-Aldrich, Burlington, MA) containing tetramethylsilane (TMS) as an internal calibration standard. Chemical shifts are reported in parts per million (ppm). The following abbreviations are used for signal multiplicity: s (singlet), d (doublet), t (triplet), q (quartet), p (pentet), m (multiplet), and br (broad). Coupling constants (*J*) are provided in hertz (Hz). The melting points (mp) were measured with open Pyrex capillary tubes and Thomas Hoover apparatus. The absorbance and fluorescence properties were measured using a Varian Cary 50 spectrophotometer (Santa Clara, CA) and Shimadzu RF-5301 PC spectrofluorometer, respectively. The VWR disposable two-sided polystyrene cuvettes with path length 1 cm were utilized to dissolve the dye in solvents for measurement. The quantum yields of dyes were measured according to the reported method with reference to indocyanine green (ICG)[1]. ESI-MS analyses were performed on a Waters Xevo G2_XS Mass Spectrometer (Waters Corporate, Milford, MA) equipped with an electrospray ionization source in positive ion mode. Each sample (5µL) was introduced into the ion source through an autosampler with 200 µL/min flow rate. The instrument operation parameters were optimized as follows: capillary voltage of 1000 V, sample cone voltage of 20 V, desolvation temperature of 350 °C, a source temperature of 120 °C. Nitrogen was used as cone gas and desolvation gas on pressures of 25 and 800 L/h, respectively. The spectra were acquired through a full scan analysis. MassLynx 4.2 software was used for data acquisition and processing. All ESI-MS spectra were acquired by Mass Spectrometry Facility at the Georgia State University Department of Chemistry.

2.2. Chemistry

2.2.1. General synthetic procedure of the indolium salts (4)

The synthesis is outlined in Scheme 1. The indolium salts were prepared according to the method developed by our lab.[2, 3] In the first step, phenylhydrazine **1** (3.0 g, 17 mmol, 1 eq) were mixed with 3-methyl-2-butanone **2** (1.8 g, 2.2 mL, 21 mmol, 1.25 eq) in acetic acid (10 mL) and refluxed in an oil bath at 110 °C for 48-72 h. The reaction was monitored using thin layer chromatography (TLC) and after the reaction completion, the reaction mixture was neutralized with saturated sodium bicarbonate solution until the effervescence stops. The mixture was then extracted using (3 x 30 mL) dichloromethane (DCM), and the combined organic layer was washed with brine, dried using anhydrous magnesium sulfate and concentrated in vacuo to obtain the indole product **3** as a red paste (85%), which was used in the following reaction without further purification. The resulting indoles **3** (3.0 g, 19 mmol, 1 eq) were mixed with various alkyl halides (3-5 eq) in acetonitrile (10 mL) and the mixture was refluxed in an oil bath at 90 °C for 24 h. The formation of the indolium salts was monitored using TLC. After the reaction completion, the reaction mixture was concentrated in vacuo, then the formed paste was crystallized using various solvent mixtures as MeOH/diethyl ether. MeOH/EtOAc, or DCM/diethyl ether to obtain the indolium salts **4** as solids with different colors (75-95%).

2.2.2. Synthesis of Vilsmeier–Haack linker of the heptamethine cyanine dyes (6)

The linker for the heptamethine cyanine dyes was obtained through the Vilsmeier-Haack chloroformylation reaction as shown in Scheme 1 and as reported[4]. Dimethyl formamide (DMF) (13 mL, 163 mmol, 3.2 eq) was dissolved in dichloromethane (13 mL) and cooled to 0 °C in an ice bath. Phosphorus oxychloride $POCl_3$ (11 mL, 115 mmol, 2.3 eq) dissolved in dichloromethane (11 mL) was added dropwise to the DMF solution while in the ice bath and stirred at 0 °C for 30 min. Cyclohexanone **5** (5.3 mL, 51 mmol, 1 eq) was added while in the ice bath, then the reaction mixture was heated in an oil bath at 70 °C for 4 h. After following up the reaction with TLC, the reaction mixture was poured on ice/water mixture (500 mL) and stirred at room temperature overnight. The formed precipitate was filtered and washed with distilled water to obtain the product **6** as a yellow solid (5.6 g, 70%).

2.2.3. General synthetic procedure for the heptamethine cyanine dyes (7)

The heptamethine cyanine dyes **7** were synthesized as shown in Scheme 1 and as reported[2, 4, 5]. The indolium salts **4** (1.0 g, 3.2 mmol, 2 eq) were condensed with the Vilsmeier linker **6** (274 mg, 1.6 mmol, 1 eq) and sodium acetate (260 mg, 3.2 mmol, 2 eq) in acetic anhydride (7 mL). The mixture was heated in an oil bath to 70 °C for 3-5 h according to the indolium salt used. The reaction was followed by using TLC and UV-vis spectroscopy. After the completion of the reaction, diethyl ether (100 mL) was added to precipitate the formed heptamethine dyes. The solid was then filtered, and the crude product was purified by various recrystallizations using either MeOH/EtOAc, MeOH diethyl ether, DCM/diethyl ether, or H_2O/acetone to obtain the heptamethine dyes **7** as green solids (68-96%).

2.2.4. General synthetic procedure for the dimeric cyanine dyes (DD1-DD8)

The dimeric cyanine dyes **DD1-8** were synthesized as shown in Schemes 2 and 3. The linkers **8a-b** (1 eq) and potassium carbonate (86 mg, 0.6 mmol, 2 eq) were mixed in ethanol (8 mL) and stirred at room temperature for 30 min. The heptamethine cyanine dyes **7** (400 mg, 0.6 mmol, 2 eq) were added and the reaction mixture was refluxed in an oil bath at 90 °C for 8 h. The reaction was monitored using TLC and UV-vis spectroscopy by observing the change in the strength of absorption bands from ~780 nm for the heptamethine monomer dyes to ~620-640 nm for the dimeric dyes. The solvent was removed in vacuo and the residue was purified by silica gel or neutral alumina flash column chromatography using DCM/MeOH then recrystallization using MeOH/diethyl ether to obtain the dimeric dyes **DD1-8** as blue solids. The synthesized dimeric dyes were characterized by ^{1}H NMR and ^{13}C NMR and were determined to be >95% pure with fair to good yields (26-49%). The HRMS was done to confirm the molecular weight of the dimeric dyes **DD1-DD8**, and all spectra showed the corresponding molecular ion peak. The positive ion mode was used for HRMS, and since the dimeric dyes **DD1-7** have two positive charges each, their *m/z* signals correspond to the molecular weight divided by two. **DD8** has six positive charges, so its *m/z* signal corresponds to the molecular weight divided by 6.

2,2'-((1E,1'E)-((3E,3'E)-(butane-1,4-diylbis(azanediyl))bis(3-(2-((E)-1-ethyl-3,3-dimethylindolin-2-ylidene)ethylidene)cyclohex-1-ene-2,1-diyl))bis(ethene-2,1-diyl))bis(1-ethyl-3,3-dimethyl-3H-indol-1-ium) iodide **DD1**. Isolated by neutral alumina column chromatography using DCM/MeOH 98:2 as a blue solid. Yield (46%, 0.14 g); mp 170-172 °C; ^{1}H NMR (400 MHz, $CDCl_3$) δ 8.12 (s, 2H), 7.72 (d, *J* = 12.8 *Hz*, 4H), 7.26 (t, *J* = 7.5 Hz , 4H), 7.21 (d, *J* = 7.5 *Hz*, 4H), 7.02 (t, *J* = 7.5 Hz, 4H), 6.84 (d, *J* = 7.5 Hz, 4H), 5.62 (d, *J* = 12.8 Hz, 4H), 3.96 (br t, 4H), 3.91 (q, *J* = 7.1 Hz, 8H), 2.52 (t, *J* = 6.8 Hz, 8H), 2.08 (br t, 4H), 1.86 (p, *J* = 6.8 Hz , 4H), 1.68 (s, 24H), 1.36 (t, *J* = 7.1 Hz, 12H); ^{13}C NMR (101 MHz, $CDCl_3$) δ 170.2, 167.1, 143.3, 140.8, 138.6, 128.5, 122.9, 122.5, 121.1, 108.5, 94.4, 50.3, 48.6, 48.3, 38.7, 29.0, 25.9, 22.0, 12.0.; HRMS (ESI) Calcd. for $[C_{72}H_{90}N_6]^{2+}$ *m/z* 519.3613, found *m/z* 519.3600. λ_{abs} = 620 nm in EtOH.

2,2'-((1E,1'E)-((3E,3'E)-(hexane-1,6-diylbis(azanediyl))bis(3-(2-((E)-1-ethyl-3,3-dimethylindolin-2-ylidene)ethylidene)cyclohex-1-ene-2,1-diyl))bis(ethene-2,1-diyl))bis(1-ethyl-3,3-dimethyl-3H-indol-1-ium) iodide **DD2**. Isolated by neutral alumina column chromatography using DCM/MeOH 98:2 as a blue solid. Yield (39%, 0.12 g); mp 168-170 °C; ^{1}H NMR (400 MHz, $CDCl_3$) δ 8.69 (s, 2H), 7.68 (d, *J* = 12.6 Hz, 4H), 7.24 (d, *J* = 7.4 Hz, 8H), 7.01 (t, *J* = 7.4 Hz, 4H), 6.82 (d, *J* = 7.4 Hz, 4H), 5.57 (d, *J* = 12.6 Hz, 4H), 3.85 (br, 12H), 2.50 (t, *J* = 6.5 Hz, 8H), 1.99 (t, *J* = 7.2 Hz, 4H), 1.82 (p, *J* = 6.5 Hz, 4H), 1.68 (s, 24H), 1.43 (br p, 4H), 1.33 (t, *J* = 6.9 Hz, 12H); ^{13}C NMR (101 MHz, CDCl3) δ 170.5, 166.5, 143.2, 137.8, 128.4, 122.9, 122.8, 122.6, 120.6, 108.3, 93.9, 50.4, 48.1, 31.2, 29.5, 26.7, 26.1, 21.9, 11.9; HRMS (ESI) Calcd. for $[C_{74}H_{94}N_6]^{2+}$ *m/z* 533.3770, found *m/z* 533.3818. λ_{abs} = 625 nm in EtOH.

2,2'-((1E,1'E)-((3E,3'E)-(butane-1,4-diylbis(azanediyl))bis(3-(2-((E)-1,3,3-trimethylindolin-2-ylidene)ethylidene)cyclohex-1-ene-2,1-diyl))bis(ethene-2,1-diyl))bis(1,3,3-trimethyl-3H-indol-1-ium) iodide **DD3.** Isolated by neutral alumina column chromatography using DCM/MeOH 97:3 as a blue solid. Yield (26%, 0.08 g); mp 159-161 °C; ^{1}H NMR (400 MHz, $CDCl_3$) δ 8.78 (s, 2H), 7.74 (br d, 4H), 7.23 (t, J = 7.8 Hz, 8H), 7.01 (d, J = 7.8 Hz, 4H), 6.83 (d, J = 7.8 Hz, 4H), 5.54 (d, J = 12.4 Hz, 4H), 3.94 (br t, 4H), 3.42 (s, 12H), 2.52 (t, J = 7.0 Hz, 8H), 2.08 (br t, 4H), 1.88 – 1.81 (m, 4H), 1.65 (s, 24H); ^{13}C NMR (101 MHz, DMSO-d_6) δ 169.0, 167.4, 143.4, 139.4, 137.4, 127.9, 122.2, 121.7, 119.9, 108.7, 94.1, 49.0, 46.9, 30.2, 28.1, 27.2, 24.7, 21.1.. λ_{abs} = 635 nm in EtOH.

2,2'-((1E,1'E)-((3E,3'E)-(butane-1,4-diylbis(azanediyl))bis(3-(2-((E)-1-isopropyl-3,3-dimethylindolin-2-ylidene)ethylidene)cyclohex-1-ene-2,1-diyl))bis(ethene-2,1-diyl))bis(1-isopropyl-3,3-dimethyl-3H-indol-1-ium) iodide **DD4.** Isolated by neutral alumina column chromatography using DCM/MeOH 98:2 as a blue solid. Yield (49%, 0.15 g); mp 171-173 °C; ^{1}H NMR (400 MHz, DMSO-d_6) δ 8.26 (s, 2H), 7.60 (d,

J = 13.0 Hz, 4H), 7.37 (d, *J* = 7.5 Hz, 4H), 7.28 (t, *J* = 7.5 Hz, 8H), 7.06 (d, *J* = 7.5 Hz, 4H), 5.88 (d, *J* = 13.0 Hz, 4H), 4.72 (septet, *J* = 6.7 Hz, 4H), 3.68 (br t, 4H), 2.47 (t, *J* = 6.2 Hz, 8H), 1.76 1.69 (m, 4H), 1.59 (br t, 4H), 1.56 (s, 24H), 1.47 (d, *J* = 6.7 Hz, 24H); ^{13}C NMR (101 MHz, DMSO-d_6) δ 168.5, 167.0, 141.5, 140.3, 138.3, 127.8, 122.1, 121.9, 119.6, 111.0, 94.8, 49.1, 46.8, 46.5, 28.4, 24.6, 21.3, 18.6, 18.5. λ_{abs} = 625 nm in EtOH.

2,2'-((1E,1'E)-((3E,3'E)-(butane-1,4-diylbis(azanediyl))bis(3-(2-((E)-1-butyl-3,3-dimethylindolin-2-ylidene)ethylidene)cyclohex-1-ene-2,1-diyl))bis(ethene-2,1-diyl))bis(1-butyl-3,3-dimethyl-3H-indol-1-ium) iodide **DD5.** Isolated by neutral alumina column chromatography using DCM/MeOH 99:1 as a blue solid. Yield (38%, 0.13 g); mp 168-170 °C; ^{1}H NMR (400 MHz, $CDCl_3$) δ 8.19 (s, 2H), 7.62 (d, *J* = 12.8 Hz, 4H), 7.17 (t, *J* = 7.7 Hz, 4H), 7.11 (d, *J* = 7.7 Hz, 4H), 6.92 (t, *J* = 7.7 Hz, 4H), 6.73 (d, *J* = 7.7 Hz, 4H), 5.53 (d, *J* = 12.8 Hz, 4H), 3.86 (br t, 4H), 3.70 (t, *J* = 7.7 Hz, 8H), 2.42 (t, *J* = 6.7 Hz, 8H), 1.99 (br t, 4H), 1.79 – 1.75 (m, 4H), 1.71 – 1.67 (m, 8H), 1.61 (s, 24H), 1.37 (p, *J* = 7.5 Hz, 8H), 0.93 (t, *J* = 7.5 Hz, 12H); ^{13}C NMR (101 MHz, $CDCl_3$) δ 170.2, 167.4, 143.8, 140.8, 138.4, 128.4, 122.8, 122.4, 121.0, 108.6, 94.7, 50.4, 48.2, 43.6, 29.8, 29.1, 29.0, 25.9, 22.1, 20.9, 14.3. λ_{abs} = 625 nm in EtOH.

2,2'-((1E,1'E)-((3E,3'E)-(butane-1,4-diylbis(azanediyl))bis(3-(2-((E)-3,3-dimethyl-1-(3-phenylpropyl)indolin-2-ylidene)ethylidene)cyclohex-1-ene-2,1-diyl))bis(ethene-2,1-diyl))bis(3,3-dimethyl-1-(3-phenylpropyl)-3H-indol-1-ium) bromide **DD6.** Isolated by neutral alumina column chromatography using DCM/MeOH 98:2 as a blue solid. Yield (43%, 0.13 g); mp 162-164 °C; ^{1}H NMR (400 MHz, DMSO-d_6) δ 8.54 (s, 2H), 7.51 (d, *J* = 13.0 Hz, 4H), 7.34 (d, *J* = 7.6 Hz, 4H), 7.32 – 7.22 (m, 20H), 7.19 (t, *J* = 7.6 Hz, 8H), 7.06 (t, *J* = 7.6 Hz, 4H), 5.47 (d, *J* = 13.0 Hz, 4H), 3.85 (t, *J* = 7.7 Hz, 8H), 3.62 (br t, 4H), 2.69 (t, *J* = 7.5 Hz, 8H), 2.21 (t, *J* = 7.7 Hz, 8H), 1.91 (p, *J* = 7.7 Hz, 8H), 1.79 (br t, 4H), 1.69 - 1.58 (m, 4H), 1.52 (s, 24H); ^{13}C NMR (101 MHz, DMSO-d_6) δ 168.7, 166.4, 142.6, 140.8, 139.5, 137.5, 128.2, 128.0, 125.8, 122.3, 121.6, 119.5, 108.8, 93.7, 46.9, 41.7, 32.0, 31.9, 28.1, 27.3, 27.3, 24.5, 21.0. λ_{abs} = 625 nm in EtOH.

2,2'-((1E,1'E)-((3E,3'E)-(butane-1,4-diylbis(azanediyl))bis(3-(2-((E)-1-(5-carboxypentyl)-3,3-dimethylindolin-2-ylidene)ethylidene)cyclohex-1-ene-2,1-diyl))bis(ethene-2,1-diyl))bis(1-(5-carboxypentyl)-3,3-dimethyl-3H-indol-1-ium) bromide **DD7.** Isolated by silica gel column chromatography using DCM/MeOH 96:4 as a blue solid. Yield (33%, 0.10 g); mp 178-180 °C; ^{1}H NMR (400 MHz, MeOD) δ 7.77 (d, *J* = 12.6 Hz, 4H), 7.37 (d, *J* = 7.5 Hz, 4H), 7.31 (t, *J* = 7.5 Hz, 4H), 7.07 (t, *J* = 7.5 Hz, 8H), 5.83 (d, *J* = 12.6 Hz, 4H), 3.96 (t, *J* = 7.4 Hz, 8H), 3.81 (t, *J* = 7.1 Hz, 4H), 3.02 (t, *J* = 7.4 Hz, 8H), 2.55 (t, *J* = 6.5 Hz, 8H), 2.25 (br p, 8H), 1.87 (br t, 4H), 1.81 (br p, 8H), 1.71 (br t, 4H), 1.68 (s, 24H), 1.49 (br p, 8H); ^{13}C NMR (101 MHz, MeOD) δ 182.5, 173.8, 173.4, 144.0, 142.6, 142.5, 129.9, 126.1, 123.9, 123.5, 112.0, 100.6, 58.5, 50.4, 45.2, 39.1, 28.8, 28.2, 28.1, 27.5, 25.5, 22.6, 18.5. λ_{abs} = 640 nm in EtOH.

2,2'-((1E,1'E)-((3E,3'E)-(butane-1,4-diylbis(azanediyl))bis(3-(2-((E)-3,3-dimethyl-1-(3-(trimethylammonio)propyl)indolin-2-ylidene)ethylidene)cyclohex-1-ene-2,1-diyl))bis(ethene-2,1-diyl))bis(3,3-dimethyl-1-(3-(trimethylammonio)propyl)-3H-indol-1-ium) bromide **DD8.** Isolated by neutral alumina column chromatography using DCM/MeOH 95:5 as a blue solid. Yield (36%, 0.13 g); mp 164-166 °C; ^{1}H NMR (400 MHz, MeOD) δ 7.73 (d, *J* = 12.8 Hz, 4H), 7.35 (t, *J* = 7.7 Hz, 8H), 7.23 (d, *J* = 7.7 Hz, 4H), 7.11 (t, *J* = 7.7 Hz, 4H), 5.93 (d, *J* = 12.8 Hz, 4H), 4.08 (t, *J* = 8.6 Hz, 8H), 3.88 (t, *J* = 7.0 Hz, 4H), 3.66 (t, *J* = 8.6 Hz, 8H), 3.21 (s, 36H), 2.66 (t, *J* = 6.5 Hz, 8H), 2.28 – 2.19 (m, 8H), 2.01 (br t, 4H), 1.85 (p, *J* = 6.5 Hz, 4H), 1.67 (s, 24H); ^{13}C NMR (101 MHz, MeOD) δ 172.3, 168.7, 144.3, 141.3, 140.1, 129.7, 124.4, 123.4, 122.7, 110.0, 96.1, 65.0, 53.9, 40.9, 29.6, 29.4, 26.7, 24.8, 21.8. λ_{abs} = 635 nm in EtOH.

2.3. Optical Studies

1 mM stock solution of each synthesized fluorophore was prepared in DMSO prior to all spectral measurements. The optical properties of these fluorophores were investigated in four solvents: ethanol (EtOH), dimethyl sulfoxide (DMSO), HEPES buffer, and phosphate-buffered saline (PBS). Absorbance spectra were acquired using a Varian Cary 50 spectrophotometer (190-1100 nm). Fluorescence emission spectra were measured on a Shimadzu RF-5301PC spectrofluorometer.

2.3.1. Quantum yield of fluorescence (Φ_f) calculation

The quantum yield of the synthesized dimeric dyes was calculated by measuring their fluorescence intensity using a Shimadzu RF-5301PC spectrofluorometer and comparing them to that of the standard FDA approved fluorophore indocyanine green (ICG) because it had a similar heptamethine cyanine structure, and because it had a comparable fluorescence wavelength (802 nm in HEPES buffer vs 803 nm for **DD1**). The values of ICG quantum yield were retrieved from the literature, and they were 14% in ethanol[6], 16.7% in DMSO[7], and 2.9% in HEPES and PBS buffers since they are water-based buffers[6]. The quantum yield of the dimeric dyes was calculated in the four different solvents used, namely: ethanol, DMSO, HEPES buffer, and PBS buffer. The dyes concentrations varied between 0.03, 0.18, or 0.36 µM. The excitation wavelength used was 600 nm, and the excitation and emission slit widths were both 5 nm.

2.4. Photothermal stability Studies

Solutions of the dimeric dyes were prepared in ethanol at a concentration of 6 µM and put in closed microwave vials. Two sets were prepared; the first set was put in dark conditions by covering them with foil plates and keeping them in a dark drawer at room temperature (25 °C), and the second set was continuously irradiated with the light of a 6000 mW 254 nm UV lamp placed 10 cm away from the dyes solutions. This second set was covered to prevent any other light from interfering and also kept at room temperature (25 °C). Samples for each dimeric dye were taken from both sets and their absorbance measured at 0 h, 24 h, 48 h, and 72 h, then the samples were returned to the vials, and stored for the next round of measurements.

2.5. Metal Sensing Studies

Stock solutions of the metal salts were prepared in 1:1 H_2O/ACN. Table S1 shows the metal salts that were used as sources of the metal ions.

Table S1. Sources of the metal ions

Source	Metal ion	Source	Metal ion
**Silver Perchlorate**	Silver I (Ag^+)	**Iron (III) Chloride**	Iron III (Fe^{3+})
**Cuprous Iodide**	Copper I (Cu^+)	**Nickel Chloride**	Nickel II (Ni^{2+})
**Lithium Chloride**	Lithium (Li^+)	**Cobalt chloride**	Cobalt II (Co^{2+})
**Sodium Chloride**	Sodium (Na^+)	**Zinc Chloride**	Zinc (Zn^{2+})
**Potassium Chloride**	Potassium (K^+)	**Mercury acetate**	Mercury II (Hg^{2+})
**Chromium Chloride**	Chromium III (Cr^{3+})	**Cupric chloride**	Copper II (Cu^{2+})

Solutions of the dimeric dye **DD1** were prepared in 50 mM HEPES buffer in 1:1 H_2O/ACN with the concentration of 10 μM, and their absorbance measured before addition of the metal solutions. Increasing amounts of metal solutions were added gradually to the dimeric dyes solutions, and the absorbance recorded after each addition. The absorbance spectra were plotted comparing the absorbance at the different concentrations of the metal ions. Absorbance spectra were acquired using a Varian Cary 50 spectrophotometer.

For the fluorescence, similar procedure was used, but the fluorescence intensity was recorded instead of the absorbance. Solutions of the dimeric dye **DD1** were prepared in 50 mM HEPES buffer in 1:1 H_2O/ACN with the concentration of 0.18 μM, and fluorescence emission spectra were measured on a Shimadzu RF-5301PC spectrofluorometer.

3. ¹H NMR, ¹³C NMR, and HRMS Spectra of the synthesized dimeric dyes

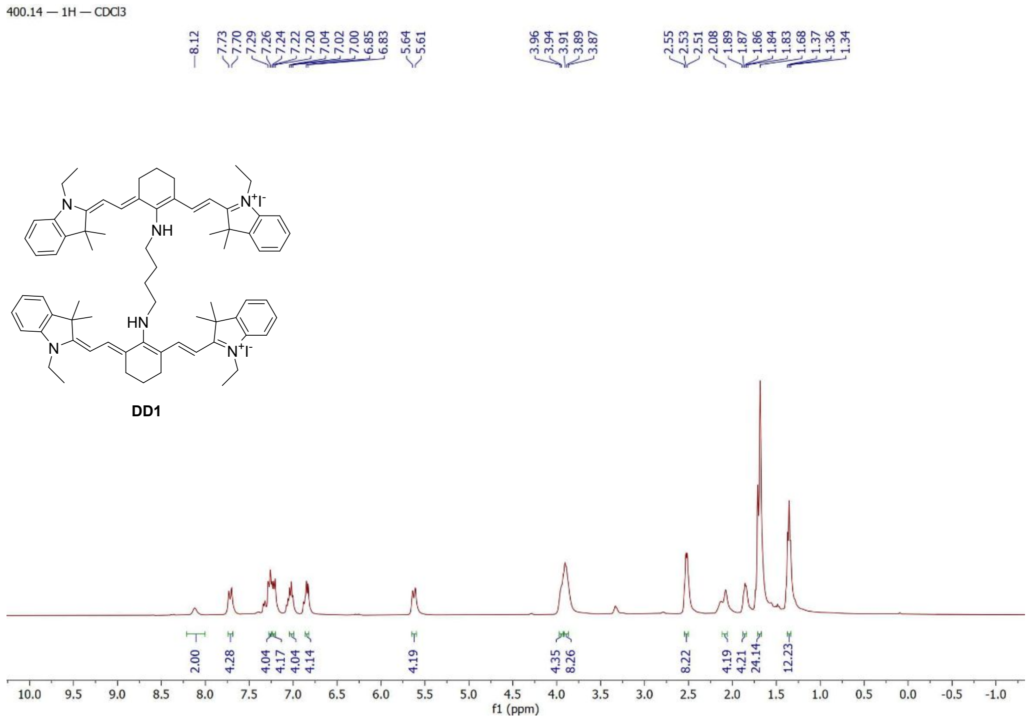

FigureS1. ¹HNMR spectrum of **DD1** in $CDCl_3$ (400 MHz)

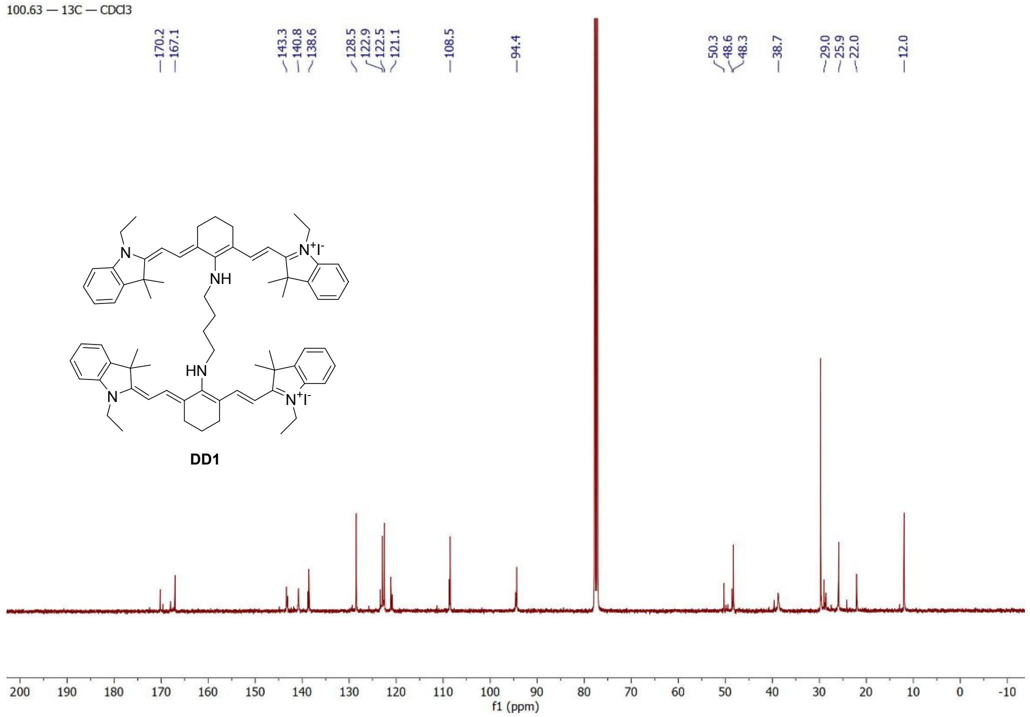

Figure S2. ¹³C NMR spectrum of **DD1** in $CDCl_3$ (101 MHz)

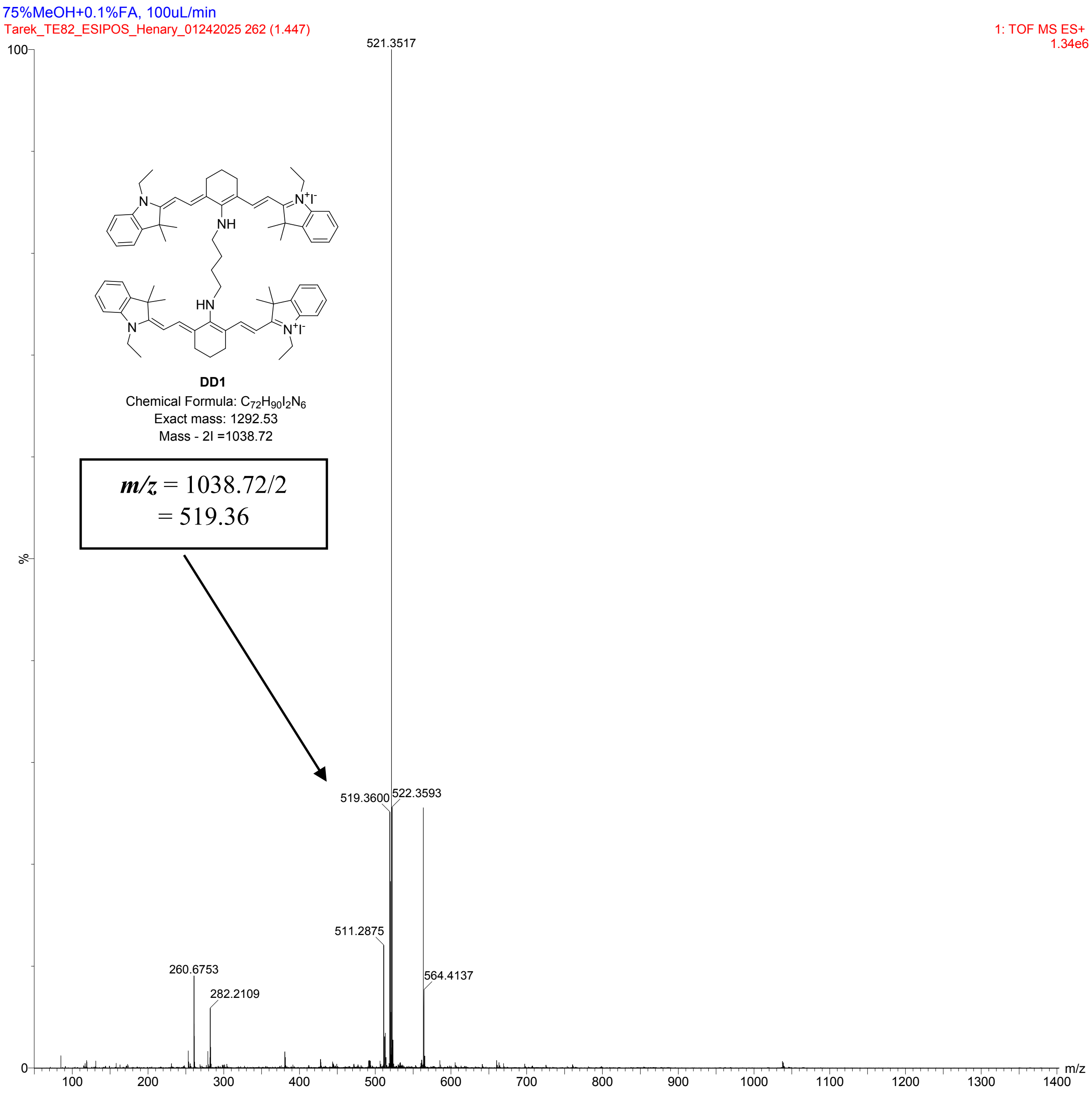

Figure S3. HRMS of **DD1**

Elemental Composition Report (DD1)

Single Mass Analysis

Tolerance = 10.0 PPM / DBE: min = -50.0, max = 500.0

Element prediction: Off

519.3600*2=1038.7200 **(Mass of DD1 -2I)**

Monoisotopic Mass, Odd and Even Electron Ions

1061 formula(e) evaluated with 2 results within limits (all results (up to 1000) for each mass)

Elements Used:

C: 72-72 H: 0-150 N: 0-25 O: 0-40

Minimum:			-50.0		
Maximum:	1000.0	10.0	500.0		
Mass	Calc. Mass	mDa	PPM	DBE	Formula
1038.7200	1038.7227	-2.7	-2.6	31.0	C72 H90 N6

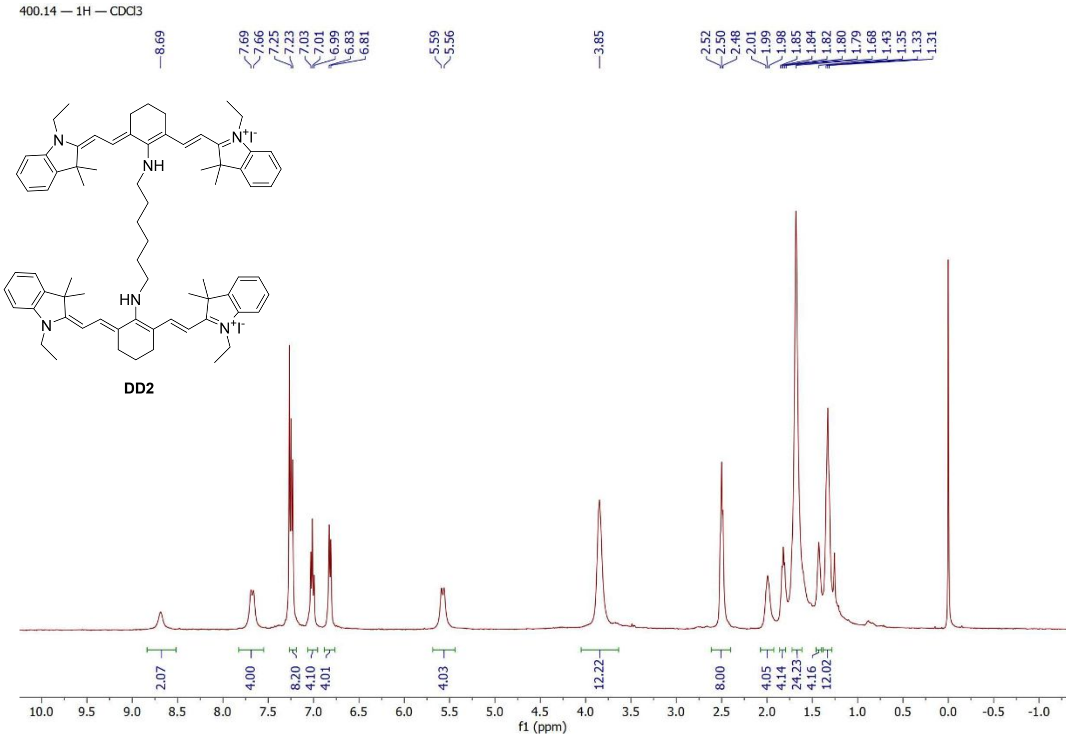

Figure S4. ^{1}HNMR spectrum of **DD2** in $CDCl_3$ (400 MHz)

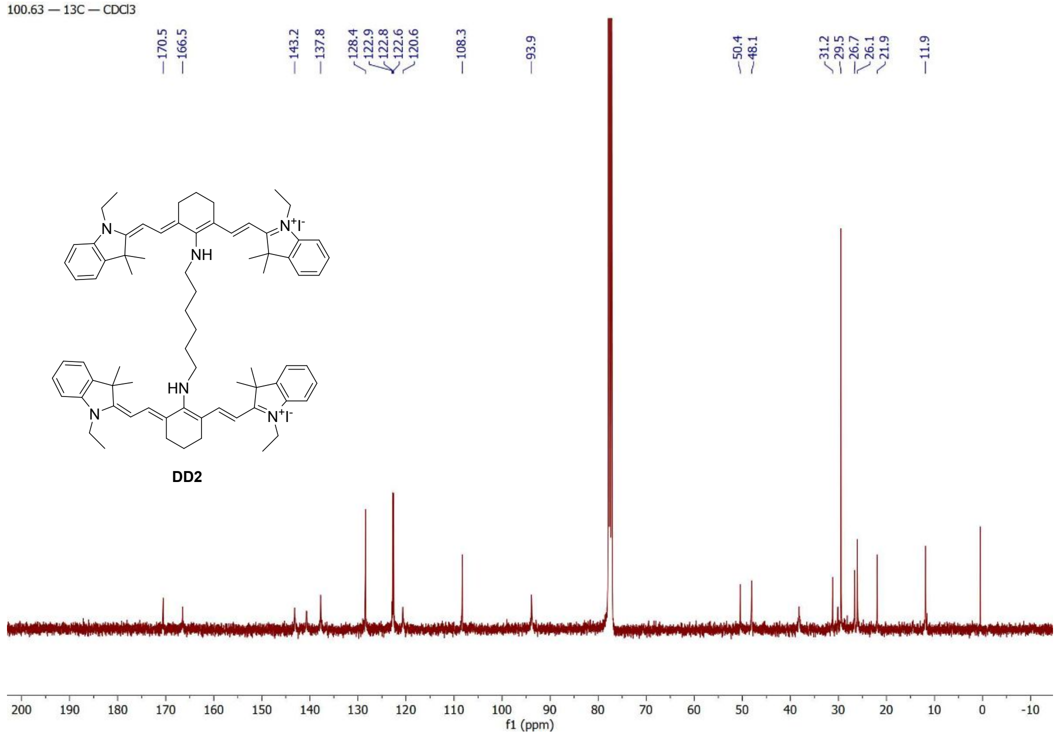

Figure S5. ^{13}C NMR spectrum of **DD2** in $CDCl_3$ (101 MHz)

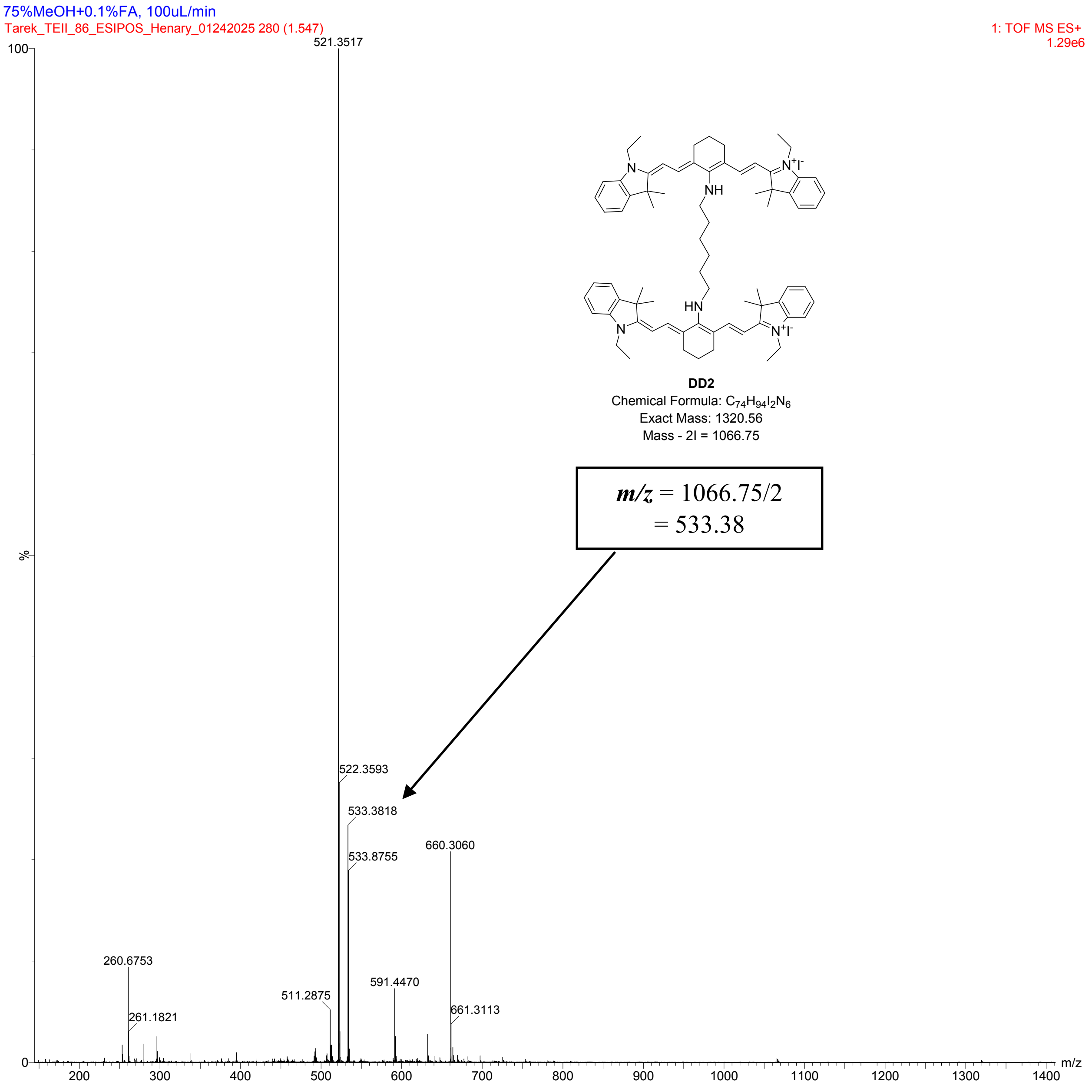

Figure S6. HRMS of **DD2**

Elemental Composition Report (DD2)

Single Mass Analysis

Tolerance = 10.0 PPM / DBE: min = -50.0, max = 500.0

Element prediction: Off

533.3818*2=1066.7636 **(Mass of DD2 – 2I)**

Monoisotopic Mass, Odd and Even Electron Ions

1059 formula(e) evaluated with 2 results within limits (all results (up to 1000) for each mass)

Elements Used:

C: 74-74 H: 0-150 N: 0-25 O: 0-40

Minimum:			-50.0			
Maximum:	1000.0	10.0	500.0			
Mass	Calc. Mass	mDa	PPM	DBE	Formula	
1066.7636	1066.7540		9.6	9.0	31.0	C74 H94 N6

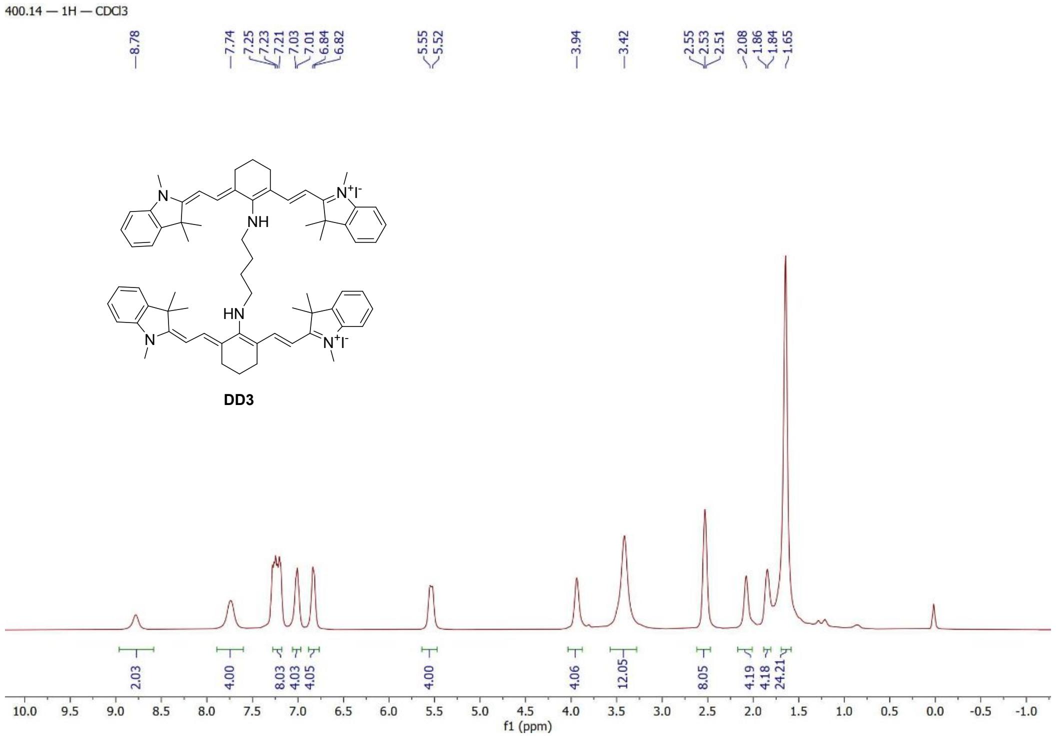

Figure S7. ^{1}HNMR spectrum of **DD3** in $CDCl_3$ (400 MHz)

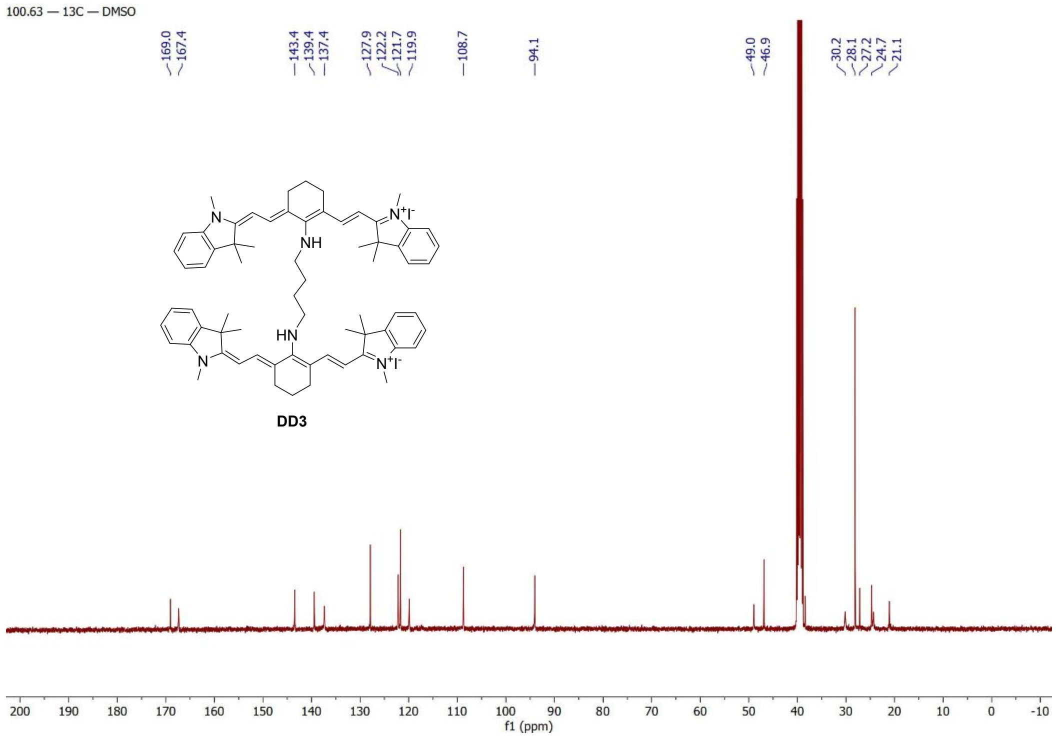

Figure S8. ^{13}C NMR spectrum of **DD3** in DMSO (101 MHz)

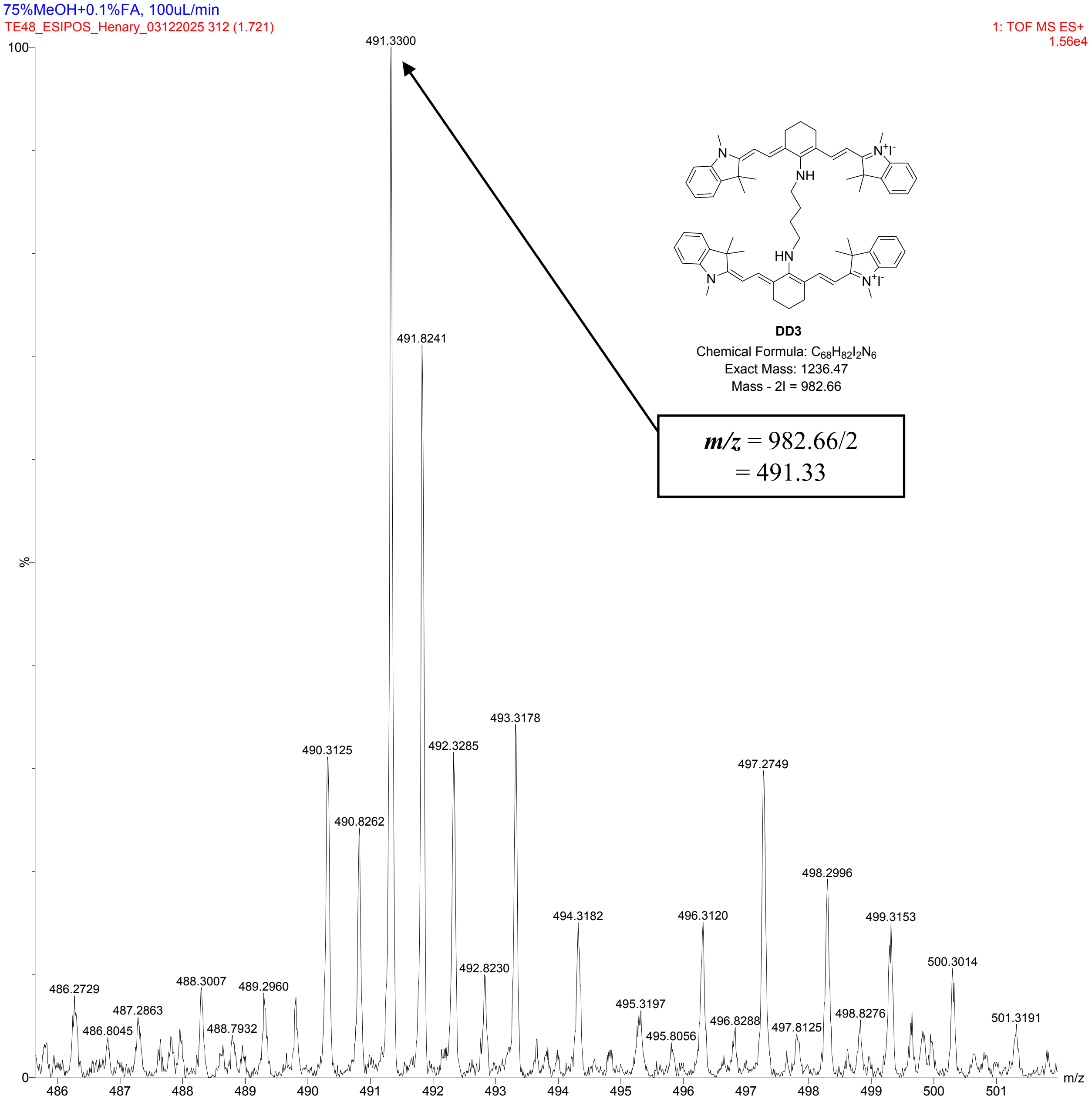

Figure S9. HRMS of **DD3**

Elemental Composition Report (DD3)

Single Mass Analysis

Tolerance = 50.0 PPM / DBE: min = -50.0, max = 500.0

Element prediction: Off

491.3300*2=982.66 **(Mass of DD3 – 2I)**

Monoisotopic Mass, Odd and Even Electron Ions

652 formula(e) evaluated with 6 results within limits (all results (up to 1000) for each mass)

Elements Used:

C: 68-68 H: 0-180 N: 0-15 O: 0-40

Minimum:			-50.0		
Maximum:	1000.0	50.0	500.0		
Mass	Calc. Mass	mDa	PPM	DBE	Formula
982.6600	982.6601	-0.1	-0.1	31.0	C68 H82 N6

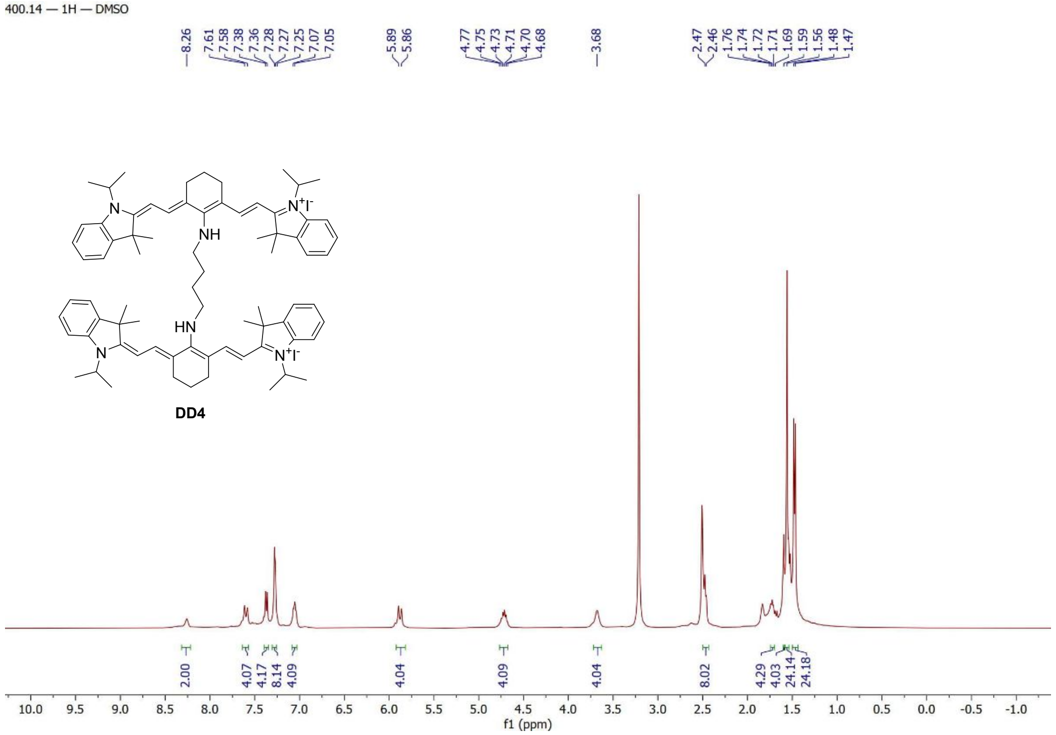

Figure S10. ^{1}HNMR spectrum of **DD4** in DMSO (400 MHz)

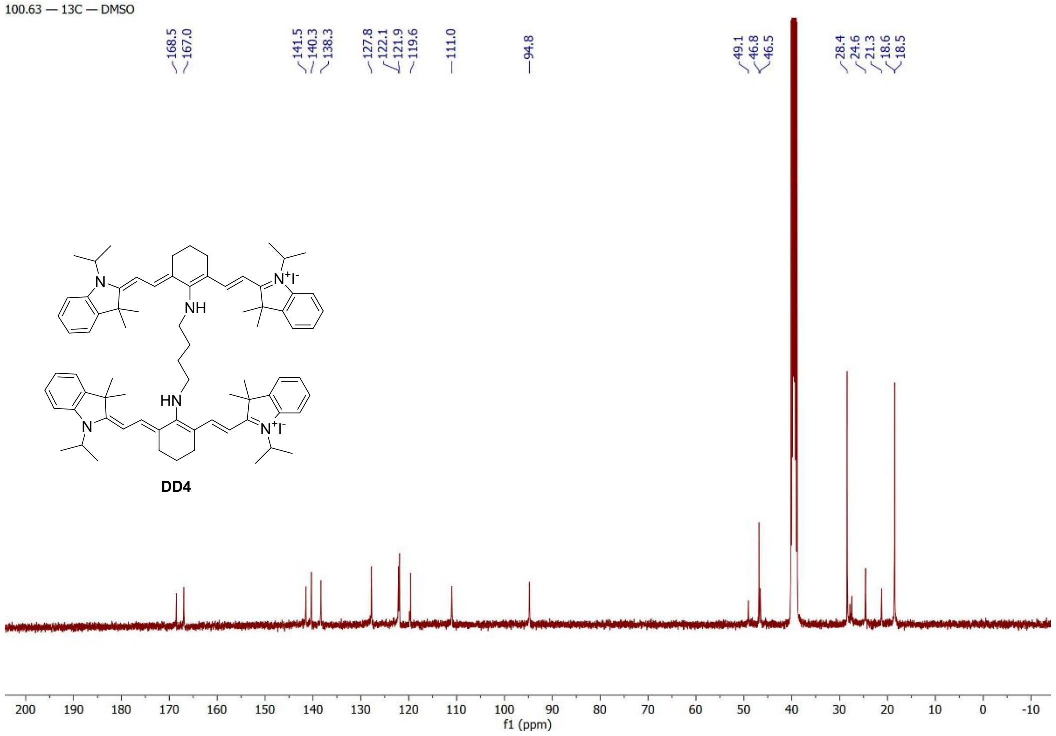

Figure S11. ^{13}C NMR spectrum of **DD4** in DMSO (101 MHz)

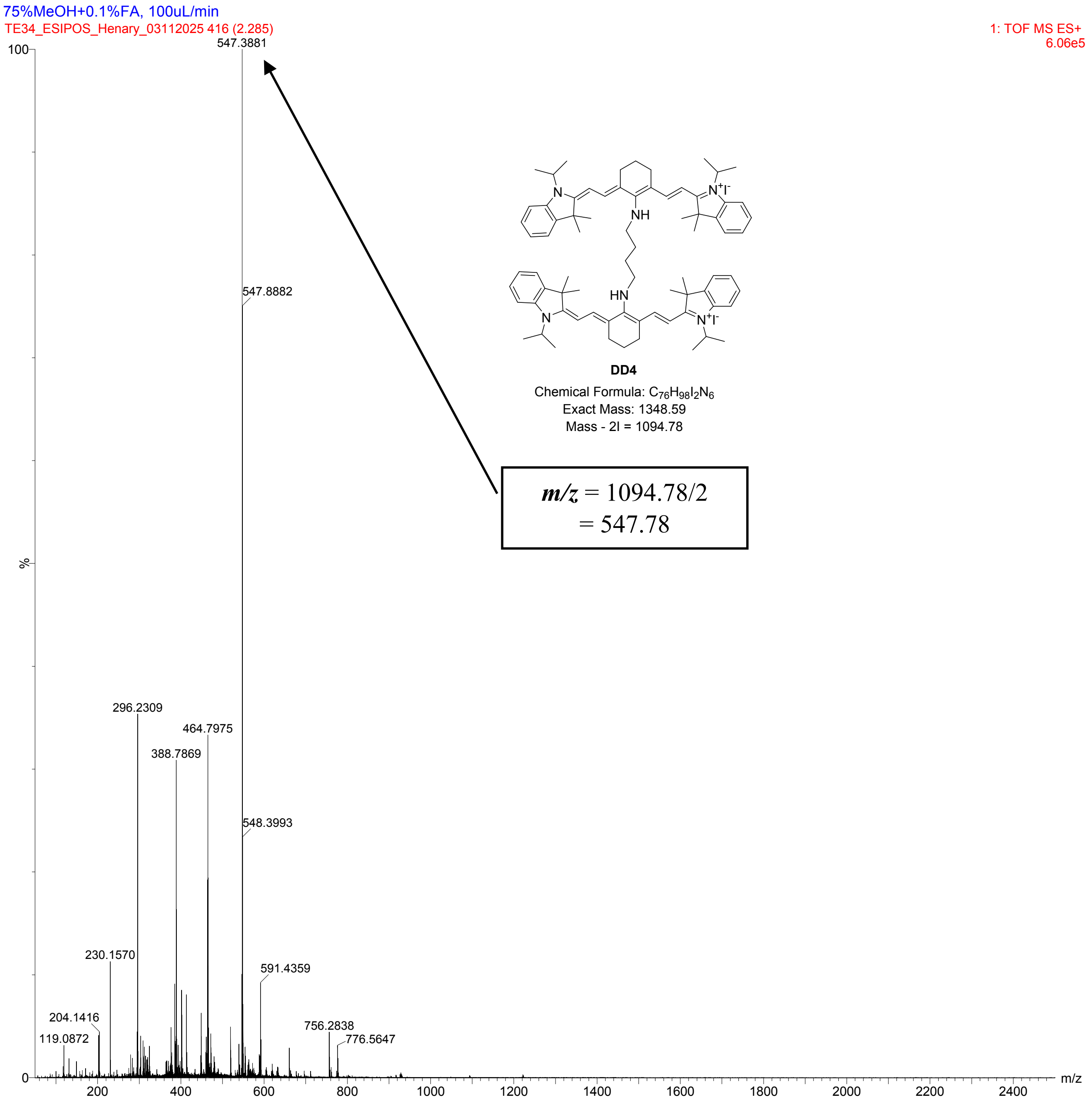

Figure S12. HRMS of **DD4**

Elemental Composition Report (DD4)

Single Mass Analysis

Tolerance = 10.0 PPM / DBE: min = -50.0, max = 500.0

Element prediction: Off

547.3881*2=1094.7762 **(Mass of DD4 -2I)**

Monoisotopic Mass, Odd and Even Electron Ions

1957 formula(e) evaluated with 3 results within limits (all results (up to 1000) for each mass)

Elements Used:

C: 76-76 H: 0-180 N: 0-15 O: 0-40 S: 0-2

Minimum:			-50.0		
Maximum:	1000.0	10.0	500.0		
Mass	Calc. Mass	mDa	PPM	DBE	Formula
1094.7762	1094.7853	-9.1	-8.3	31.0	C76 H98 N6

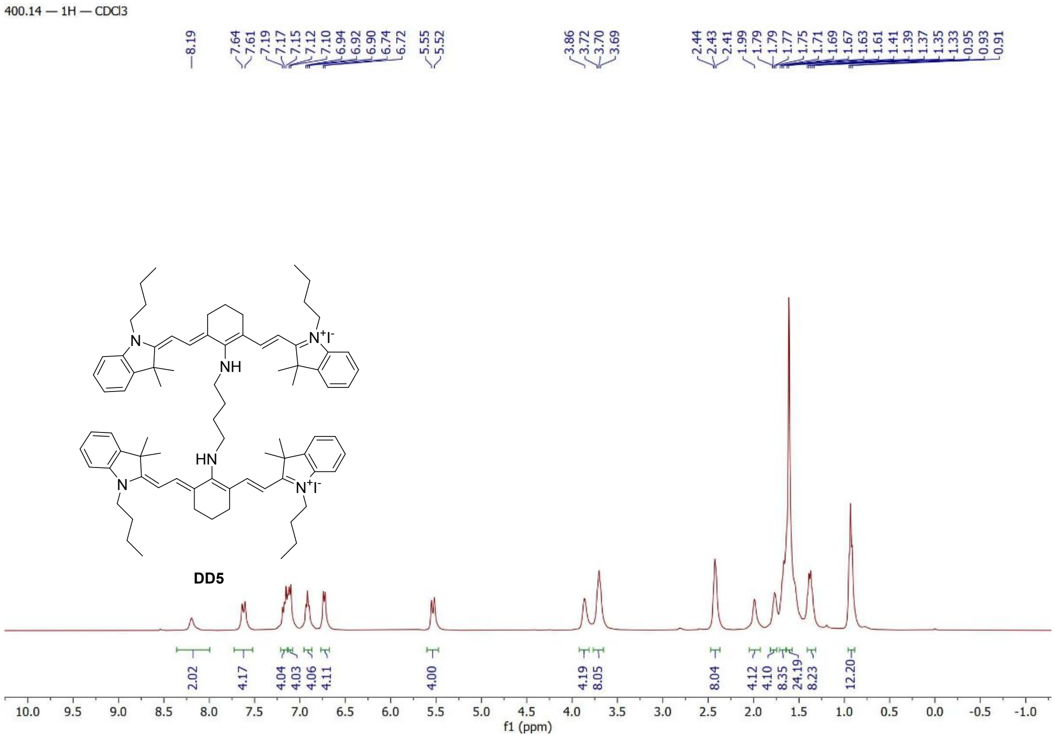

Figure S13. ¹HNMR spectrum of **DD5** in $CDCl_3$ (400 MHz)

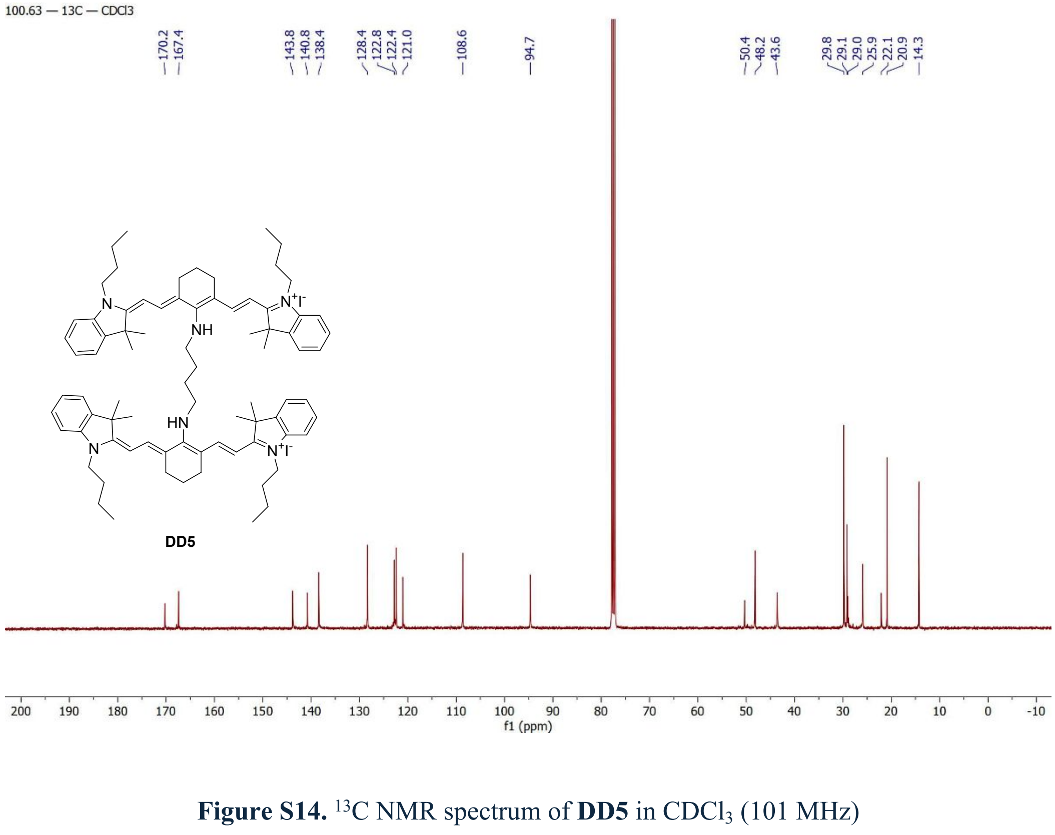

Figure S14. ¹³C NMR spectrum of **DD5** in $CDCl_3$ (101 MHz)

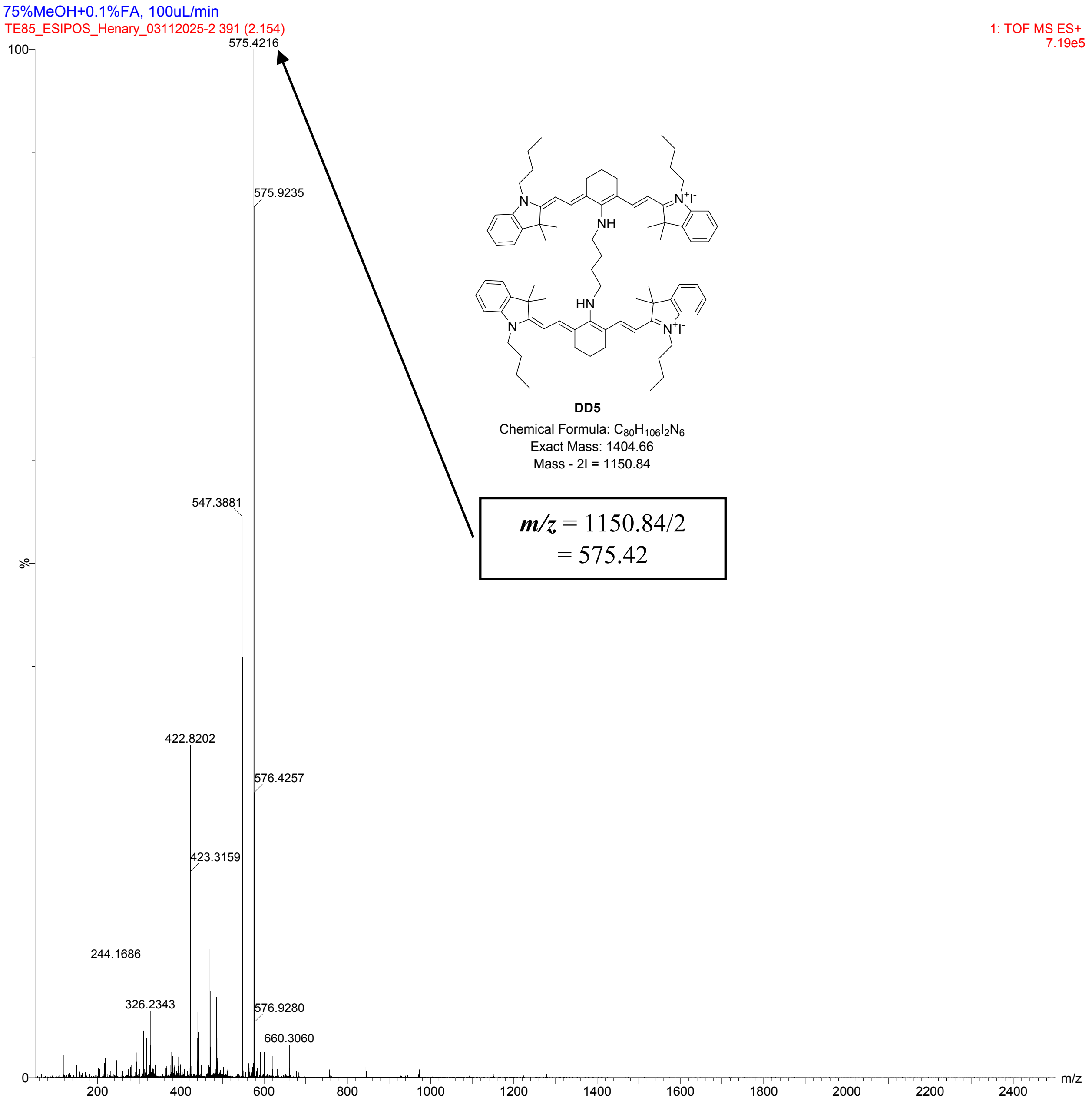

FigureS15. HRMS of **DD5**

Elemental Composition Report (DD5)

Single Mass Analysis

Tolerance = 10.0 PPM / DBE: min = -50.0, max = 500.0

Element prediction: Off

575.4216*2=1150.8432 **(Mass of DD5 -2I)**

Monoisotopic Mass, Odd and Even Electron Ions

1960 formula(e) evaluated with 3 results within limits (all results (up to 1000) for each mass)

Elements Used:

C: 80-80 H: 0-180 N: 0-15 O: 0-40 S: 0-2

Minimum:			-50.0		
Maximum:	1000.0	10.0	500.0		
Mass	Calc. Mass	mDa	PPM	DBE	Formula
1150.8432	1150.8479	-4.7	-4.1	31.0	C80 H106 N6

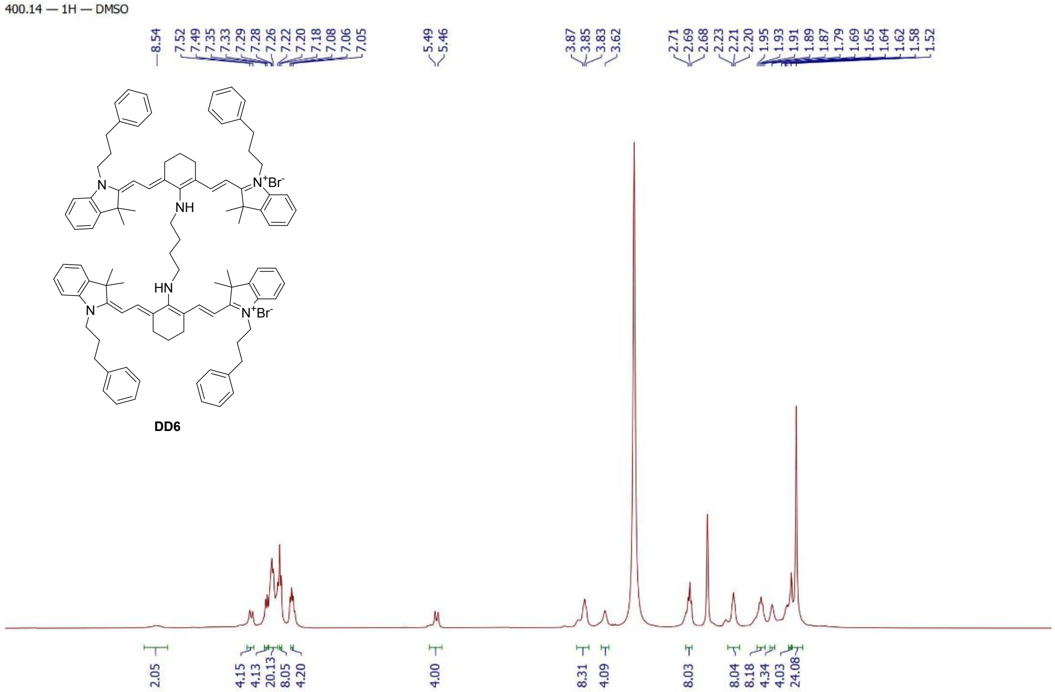

Figure S16. ^{1}HNMR spectrum of **DD6** in DMSO (400 MHz)

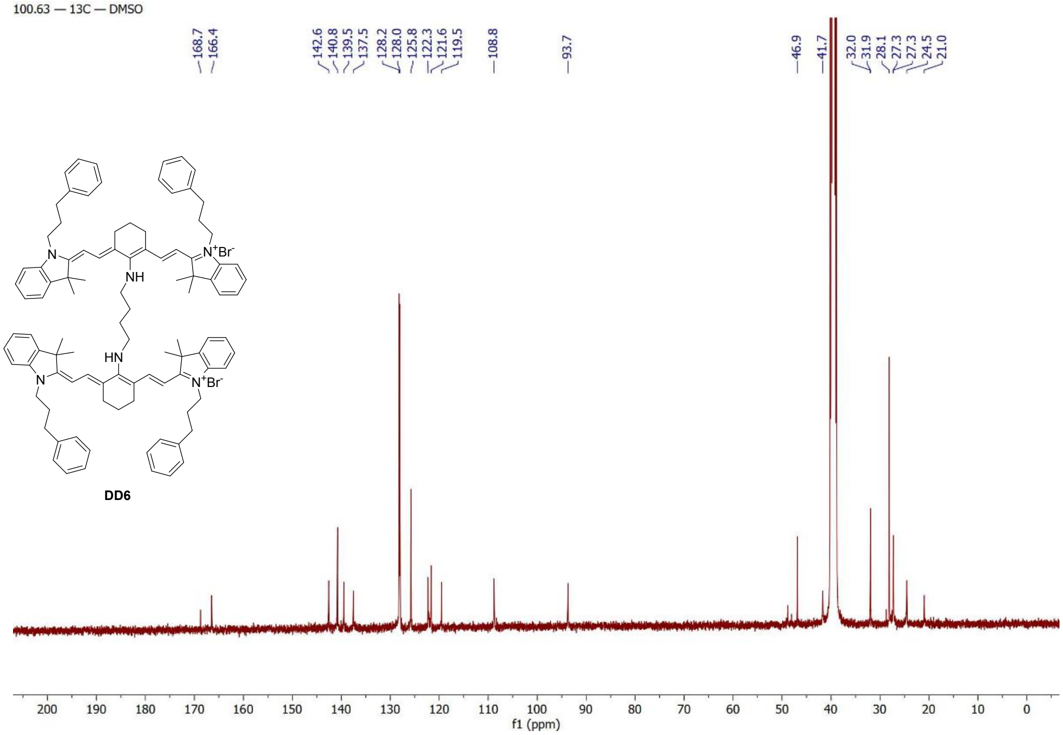

Figure S17. ^{13}C NMR spectrum of **DD6** in DMSO (101 MHz)

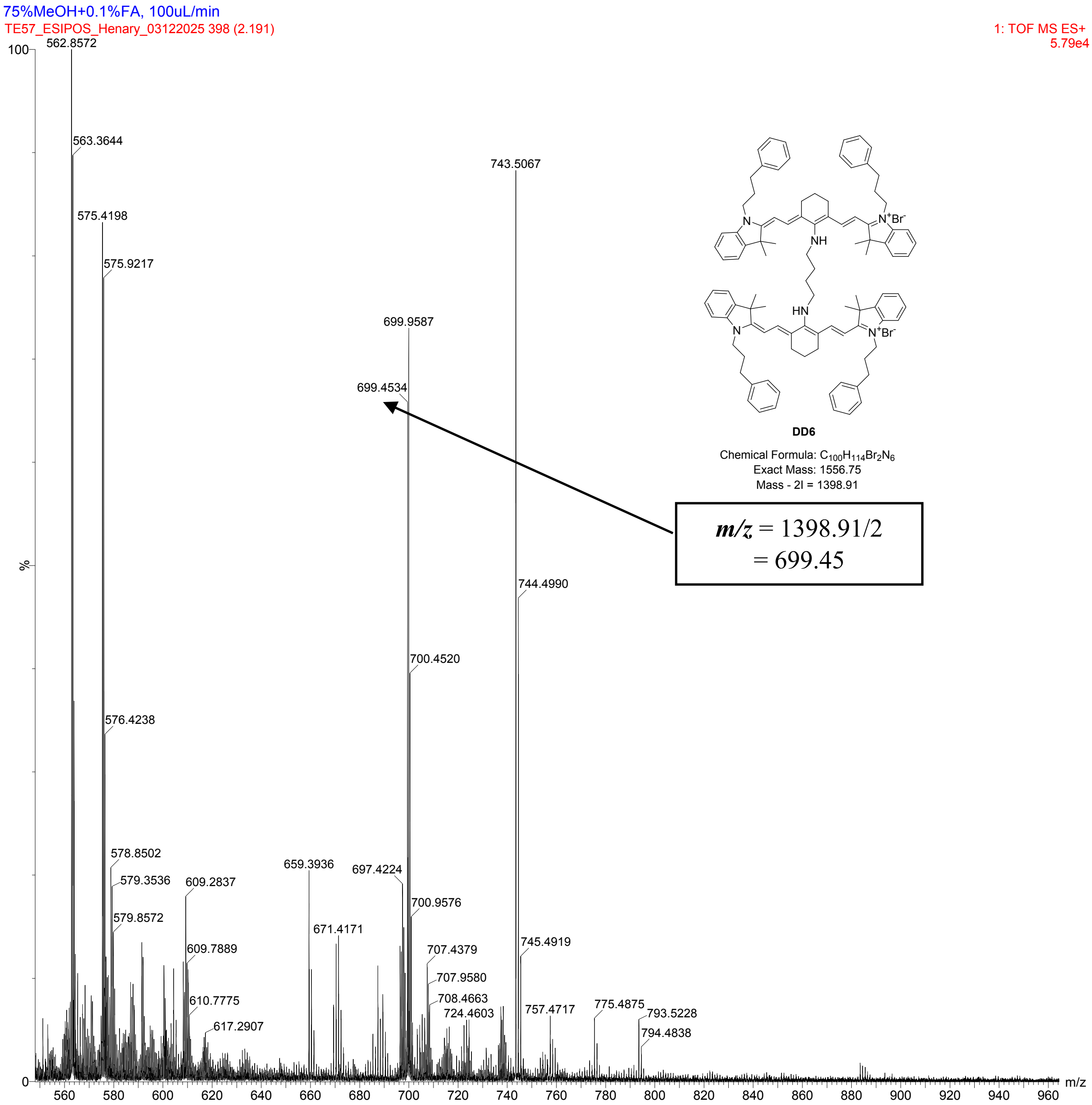

Figure S18. HRMS of **DD6**

Elemental Composition Report (DD6)

Single Mass Analysis

Tolerance = 20.0 PPM / DBE: min = -50.0, max = 500.0

Element prediction: Off

699.4534*2=1398.9068 **(Mass of DD6 -2Br)**

Monoisotopic Mass, Odd and Even Electron Ions

649 formula(e) evaluated with 4 results within limits (all results (up to 1000) for each mass)

Elements Used:

C: 100-100 H: 0-180 N: 0-15 O: 0-40

Minimum:			-50.0		
Maximum:	1000.0	20.0	500.0		
Mass	Calc. Mass	mDa	PPM	DBE	Formula
1398.9068	1398.9105	-3.7	-2.6	47.0	C100 H114 N6

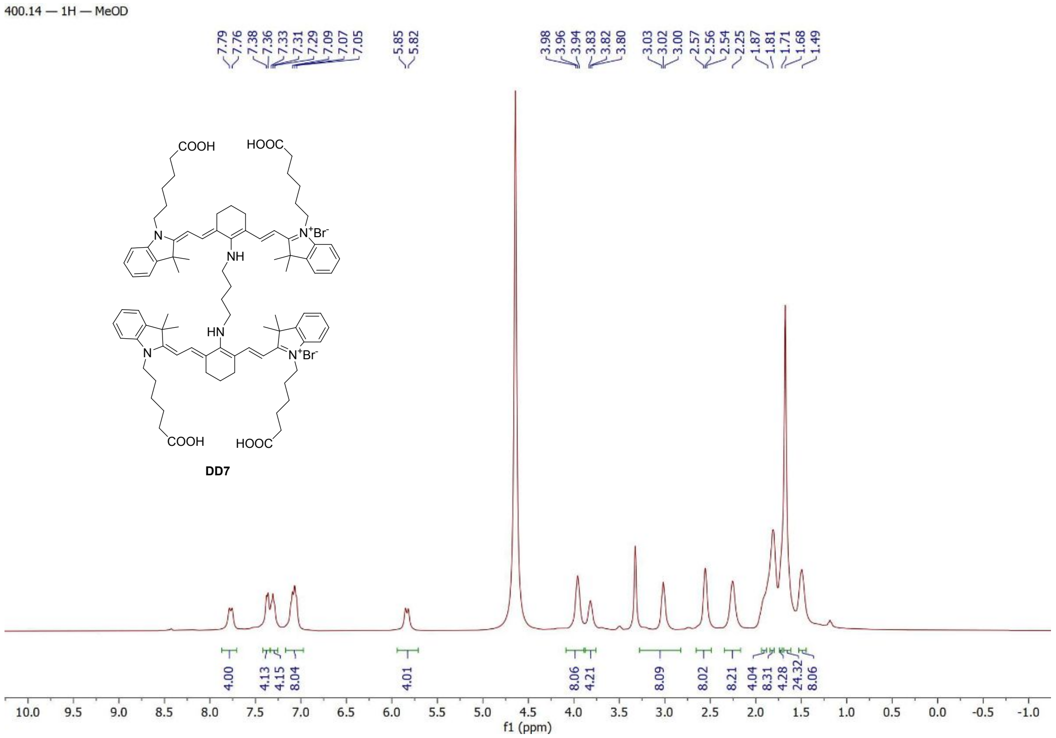

Figure S19. ^{1}HNMR spectrum of **DD7** in MeOD (400 MHz)

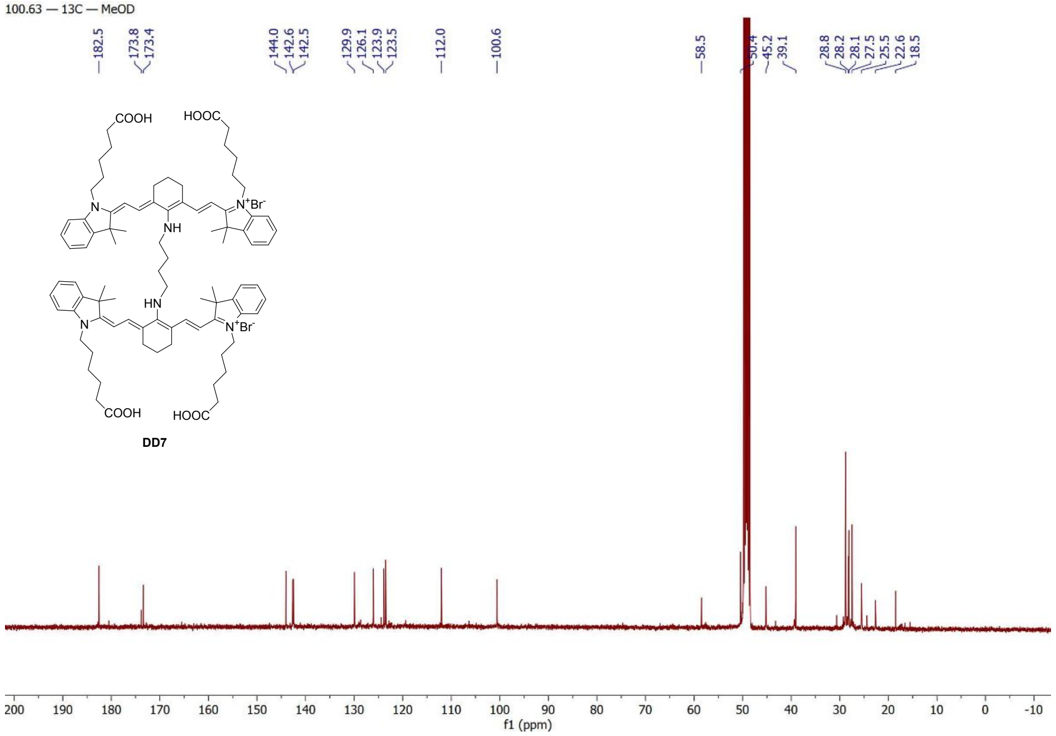

Figure S20. ^{13}C NMR spectrum of **DD7** in MeOD (101 MHz)

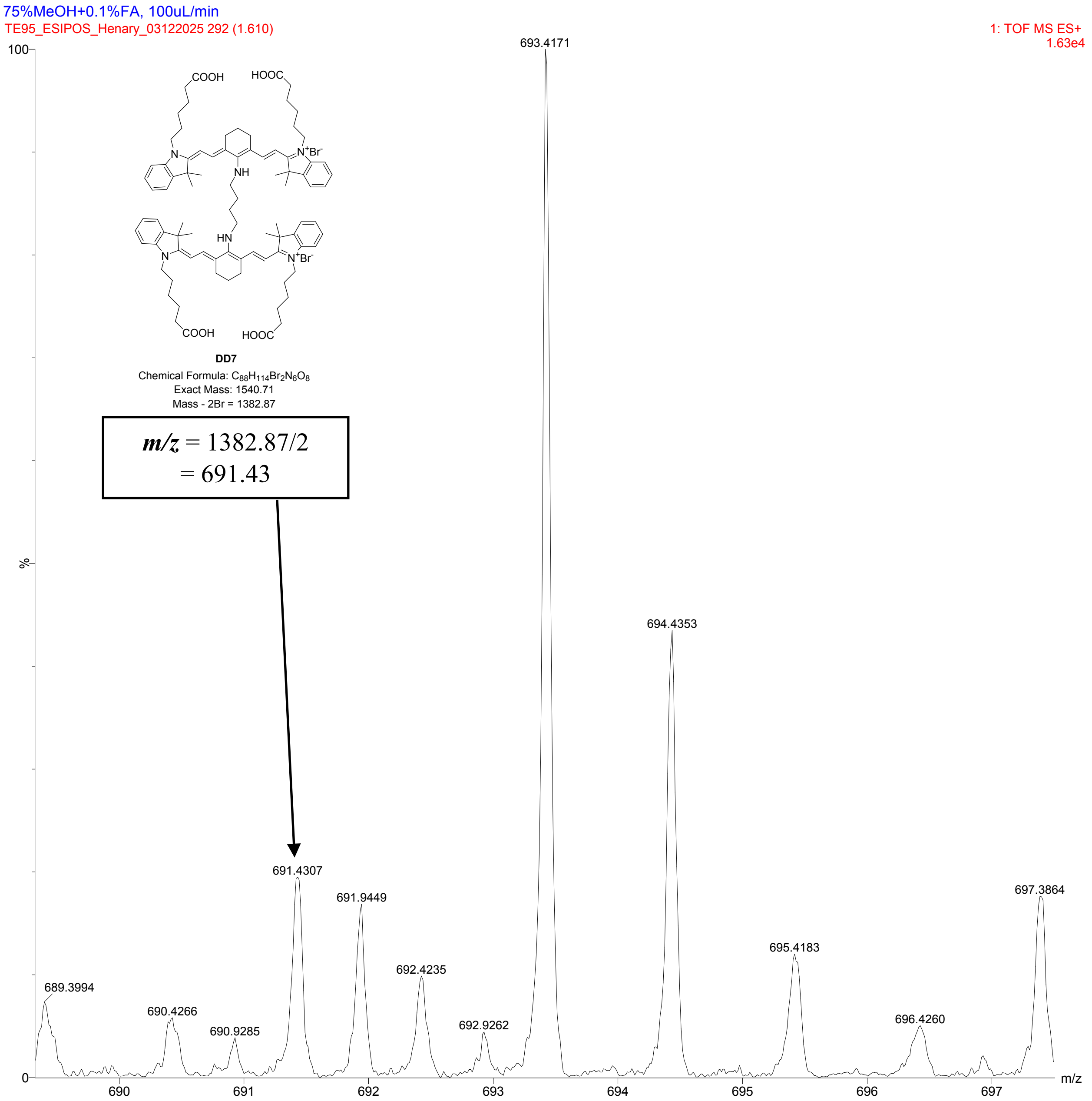

Figure S21. HRMS of **DD7**

Elemental Composition Report (DD7)

Single Mass Analysis

Tolerance = 10.0 PPM / DBE: min = -50.0, max = 500.0

Element prediction: Off

691.4307*2=1382.8614 **(Mass of DD7 -2Br)**

Monoisotopic Mass, Odd and Even Electron Ions

985 formula(e) evaluated with 3 results within limits (all results (up to 1000) for each mass)

Elements Used:

C: 88-88 H: 0-150 N: 0-25 O: 0-40

Minimum:			-50.0		
Maximum:	1000.0	10.0	500.0		
Mass	Calc. Mass	mDa	PPM	DBE	Formula
1382.8614	1382.8698	-8.4	-6.1	35.0	C88 H114 N6 O8

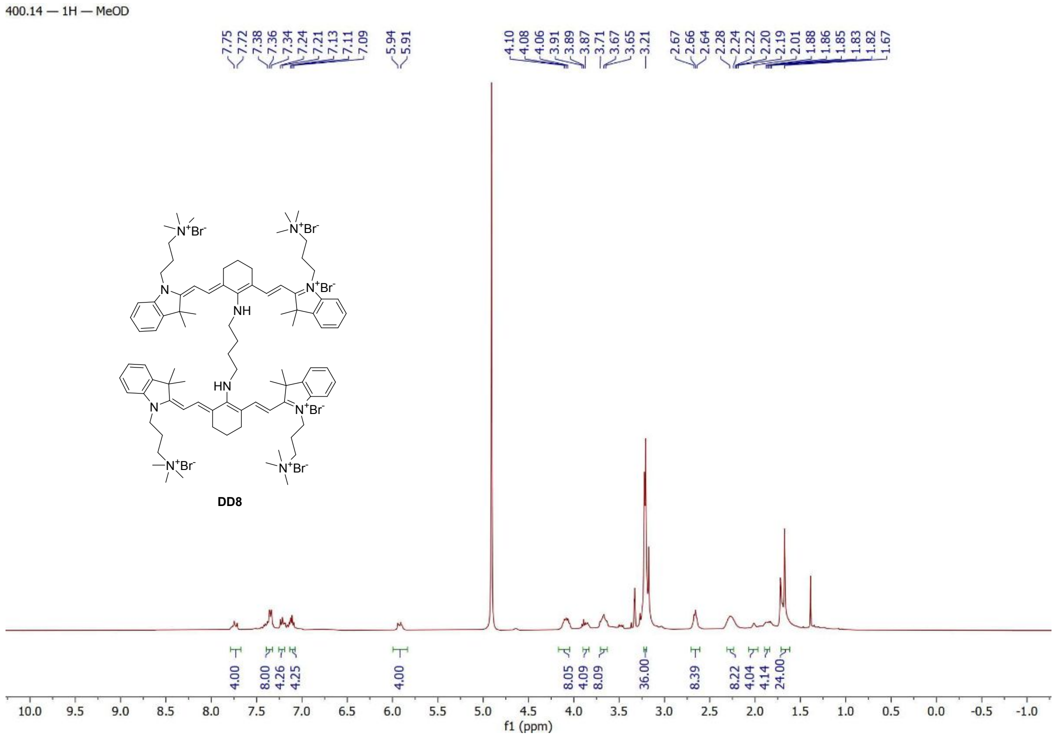

Figure S22. ^{1}HNMR spectrum of **DD8** in MeOD (400 MHz)

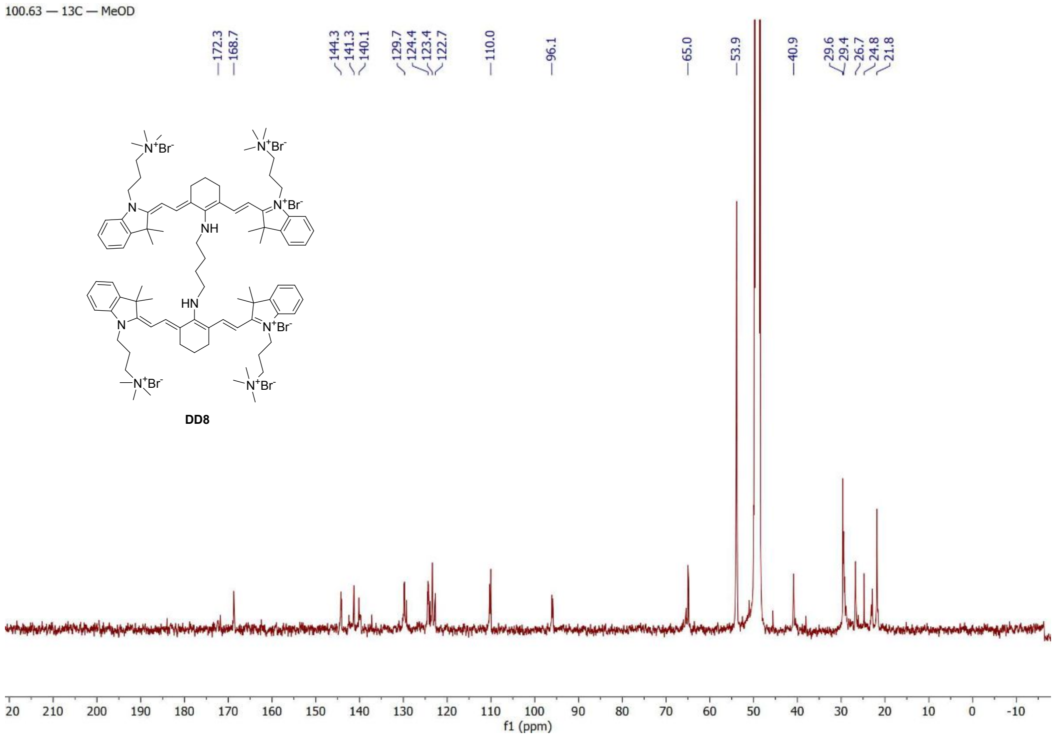

Figure S23. ^{13}C NMR spectrum of **DD8** in MeOD (101 MHz)

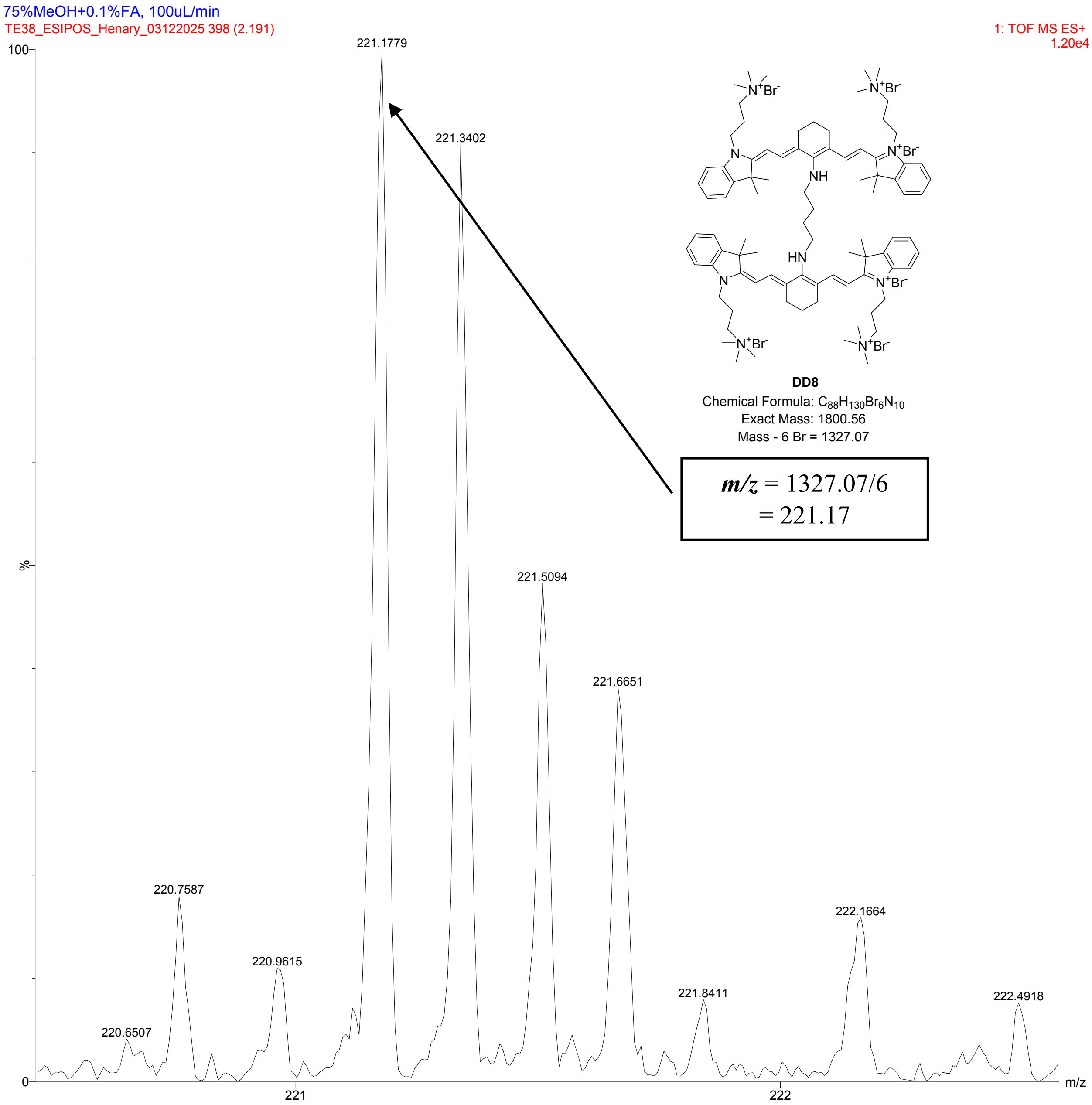

Figure S24. HRMS of **DD8**

Elemental Composition Report (DD8)

Single Mass Analysis

Tolerance = 50.0 PPM / DBE: min = -50.0, max = 500.0

Element prediction: Off

221.1779*6=1327.0674 **(Mass of DD8 -2Br)**

Monoisotopic Mass, Odd and Even Electron Ions

628 formula(e) evaluated with 27 results within limits (all results (up to 1000) for each mass)

Elements Used:

C: 88-88 H: 0-180 N: 0-15 O: 0-40

Minimum:			-20.0		
Maximum:	1000.0	20.0	500.0		
Mass	Calc. Mass	mDa	PPM	DBE	Formula
1327.0674	1327.0480	19.4	14.6	29.0	C88 H130 N10

4. Physicochemical Properties of the synthesized dimeric dyes

Table S2. Physicochemical properties of the synthesized dimeric dyes as predicted by Chemaxon® Marvinsketch

Dye	MW	Log D	Rotatable bonds	Surface Area (Å^2)	TPSA (Å^2)	Volume (Å^3)	HBD/HBA	Polarizability
**DD1**	1,293.36	6.43	17	1,668.15	36.56	1,042.97	2/4	127.95
**DD2**	1,321.42	7.30	19	1,805.22	36.56	1,076.93	2/4	131.64
**DD3**	1,237.26	5.00	13	1,542.73	36.56	974.10	2/4	120.56
**DD4**	1,349.47	8.08	17	1,786.48	36.56	1,110.13	2/4	135.34
**DD5**	1,405.58	10.29	25	1,917.04	36.56	1,181.21	2/4	142.72
**DD6**	1,559.86	14.83	29	2,210.03	36.56	1,393.60	2/4	173.58
**DD7**	1,543.72	7.90	37	2,187.36	185.76	1,360.65	6/12	159.23
**DD8**	1,807.50	-11.33	29	2,280.53	36.56	1,383.04	2/4	163.27

Log D: Distribution coefficient was calculated at pH = 7.4, MW: Molecular Weight, TPSA: total polar surface area, HBD: H-bond donor, HBA: H-bond acceptor

5. Tables of optical properties in different solvents

Table S3: Optical properties of ICG in HEPES buffer

Dye	Absorbance Wavelength Maxima (nm)	Excitation Wavelength (nm)	Emission Wavelength (nm)	Stokes Shift (nm)	Extinction coefficient ($M^{-1}cm^{-1}$)	Quantum Yield of Fluorescence (%)	Molecular Brightness ($M^{-1}cm^{-1}$)
**ICG**	778	720	802	24	148,000	2.9[6]	4,292
**ICG from literature[6]**	779	710	805	26	156,000	2.9	4,500

Table S4: Optical properties of the dimeric dyes in ethanol

Dye	Absorbance Wavelength Maxima (nm)	Excitation Wavelength (nm)	Emission Wavelength (nm)	Stokes Shift (nm)	Extinction coefficient ($M^{-1}cm^{-1}$)	Quantum Yield of Fluorescence (%)	Molecular Brightness ($M^{-1}cm^{-1}$)
**DD1**	620	600	751	131	125,100	12.6	15,740
**DD2**	625	600	749	124	116,400	29.3	34,075
**DD3**	635	600	748	113	75,800	43.9	33,309
**DD4**	625	600	753	128	120,500	14.2	17,092
**DD5**	625	600	750	125	121,800	15.0	18,240
**DD6**	625	600	755	130	84,700	22.2	18,834
**DD7**	640	600	747	107	36,000	11.9	4,275
**DD8**	635	600	740	105	123,000	25.9	31,908
**ICG**	785	710	825	40	215,000	14.0[6]	30,100
**ICG from literature[6]**	787	710	815	28	223,000	14.0	31,000

Table S5: Optical properties of the dimeric dyes in DMSO

Dye	Absorbance Wavelength Maxima (nm)	Excitation Wavelength (nm)	Emission Wavelength (nm)	Stokes Shift (nm)	Extinction coefficient ($M^{-1}cm^{-1}$)	Quantum Yield of Fluorescence (%)	Molecular Brightness ($M^{-1}cm^{-1}$)
**DD1**	630	600	759	129	130,100	23.6	30,762
**DD2**	625	600	760	135	118,200	41.1	48,569
**DD3**	625	600	759	134	66,100	42.3	27,934
**DD4**	635	600	761	126	90,900	27.2	24,699
**DD5**	625	600	759	134	114,600	11.6	13,243
**DD6**	625	600	758	133	68,300	20.8	14,236
**DD7**	630	600	767	137	28,300	38.9	11,013
**DD8**	635	600	758	123	95,000	37.1	35,288
**ICG**	798	720	817	19	216,000	16.7[7]	36,072
**ICG from literature[7]**	795	730	820	25	224,000	16.7	37,000

Table S6: Optical properties of the dimeric dyes in PBS buffer

Dye	Absorbance Wavelength Maxima (nm)	Excitation Wavelength (nm)	Emission Wavelength (nm)	Stokes Shift (nm)	Extinction coefficient ($M^{-1}cm^{-1}$)	Quantum Yield of Fluorescence (%)	Molecular Brightness ($M^{-1}cm^{-1}$)
**DD1**	625	600	759	134	59,300	3.2	1,894
**DD2**	625	600	758	133	54,300	3.8	2,079
**DD3**	625	600	754	129	97,300	3.4	3,344
**DD4**	625	600	759	134	62,400	1.3	827
**DD5**	615	600	771	156	45,400	2.1	960
**DD6**	615	600	787	172	47,900	1.1	514
**DD7**	635	600	764	129	16,700	9.8	1,635
**DD8**	600	600	758	158	73,800	3.8	2,808
**ICG**	778	720	802	24	146,000	2.9[6]	4,234
**ICG from literature[6]**	779	710	805	26	156,000	2.9	4,500

6. Absorbance and Emission Spectra of the synthesized dimeric dyes

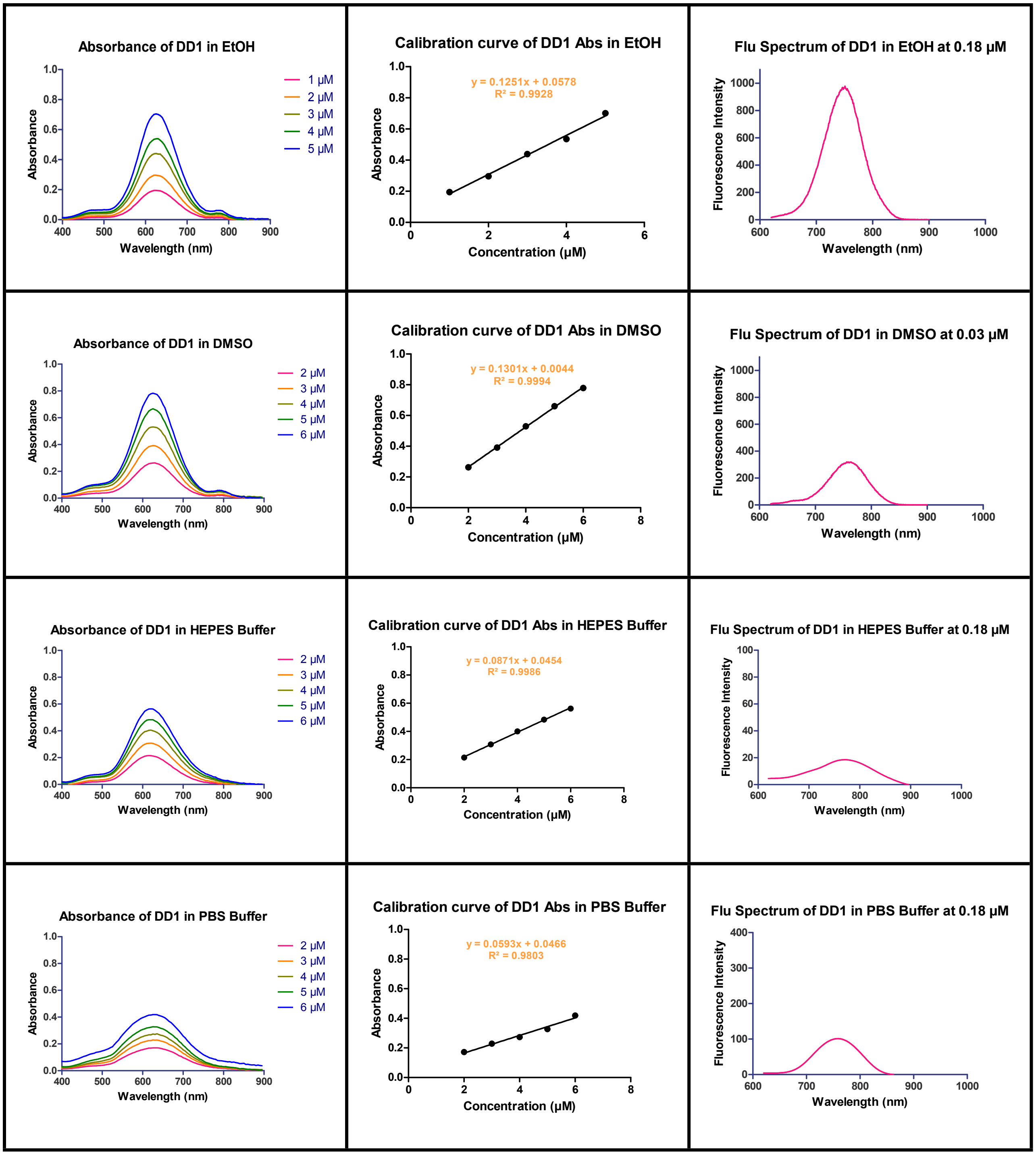

Figure S25. DD1 Absorbance curves at different concentrations, calibration curves, and fluorescence curves in different solvents

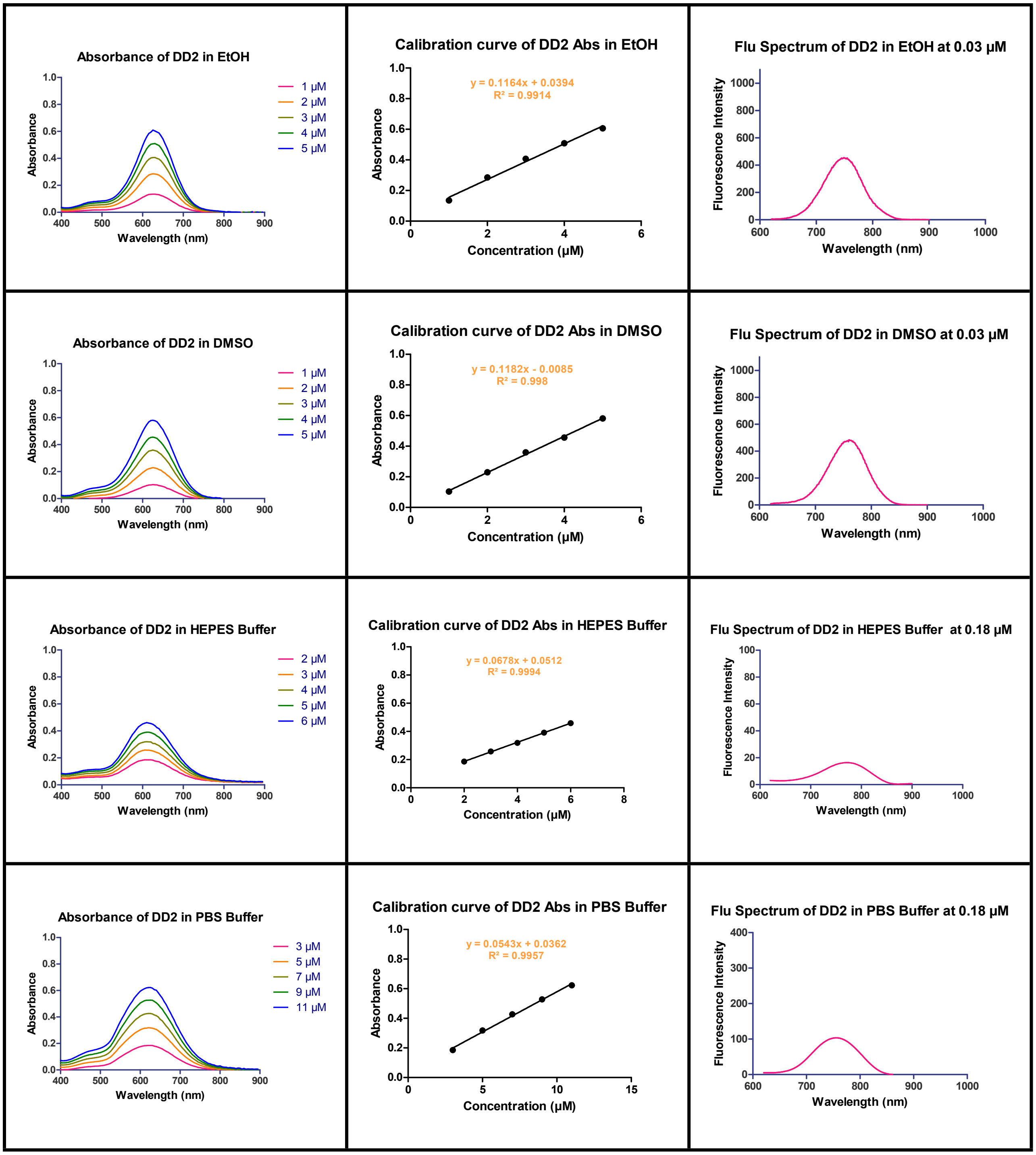

Figure S26. DD2 Absorbance curves at different concentrations, calibration curves, and fluorescence curves in different solvents

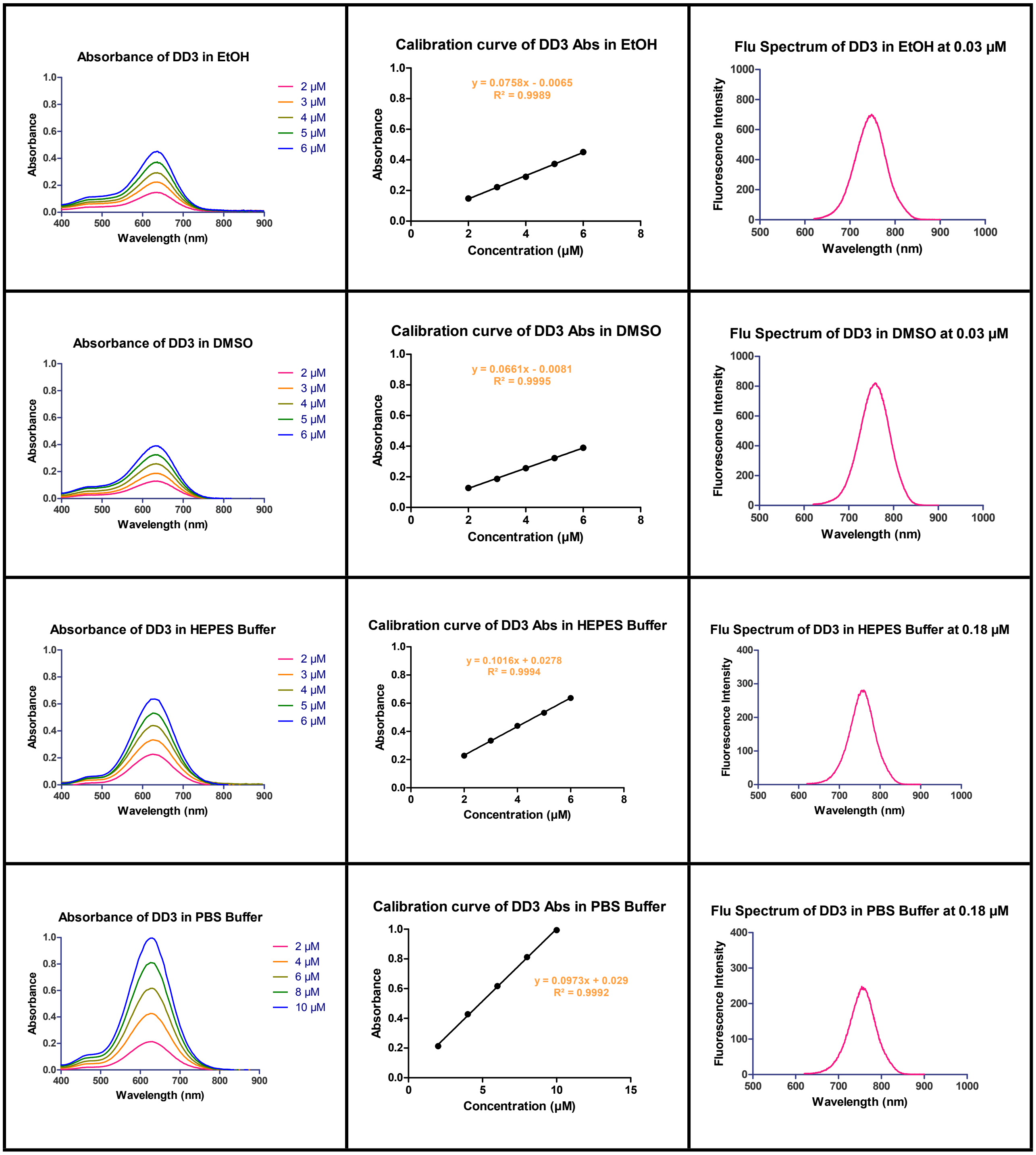

Figure S27. DD3 Absorbance curves at different concentrations, calibration curves, and fluorescence curves in different solvents

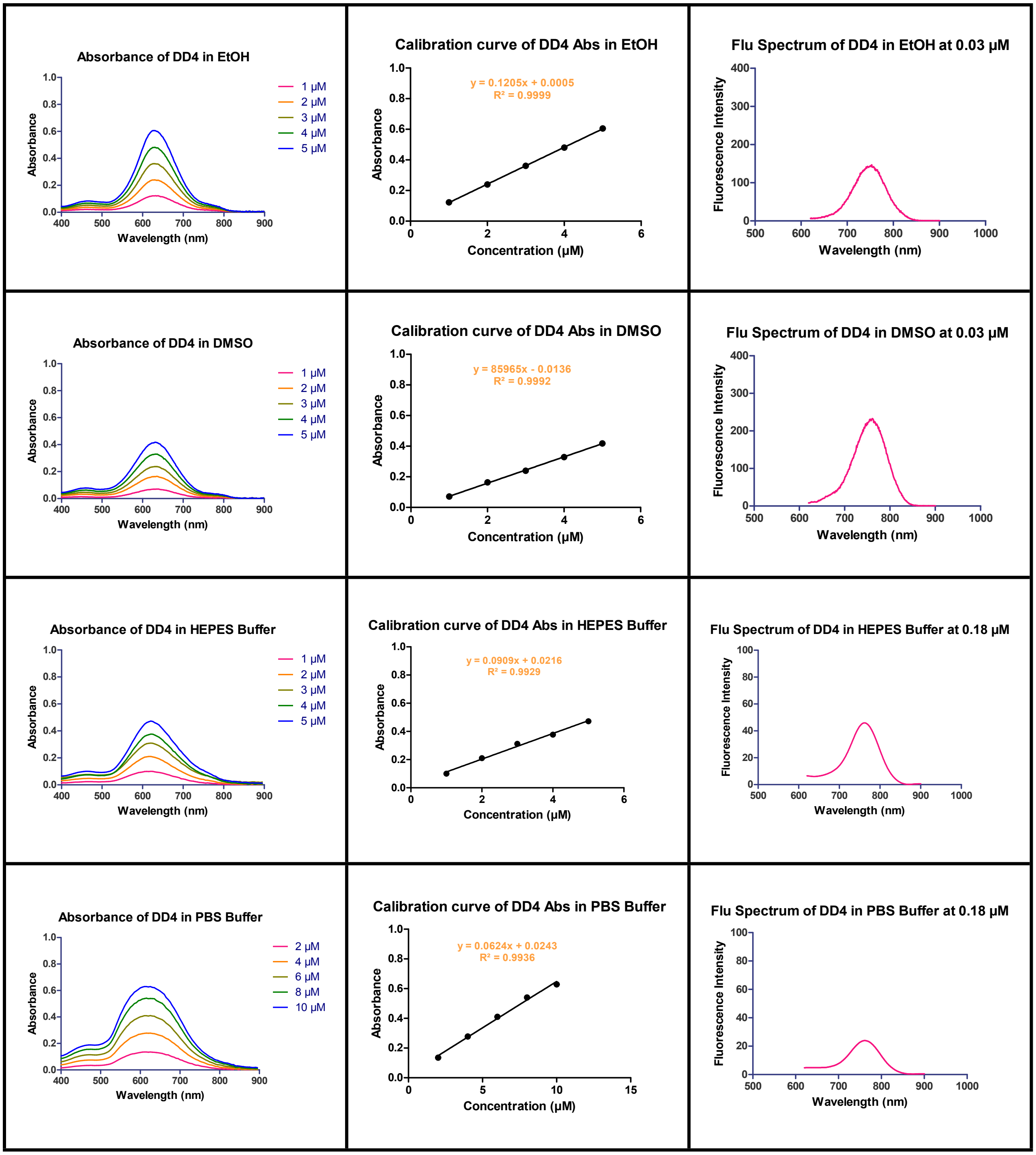

Figure S28. DD4 Absorbance curves at different concentrations, calibration curves, and fluorescence curves in different solvents

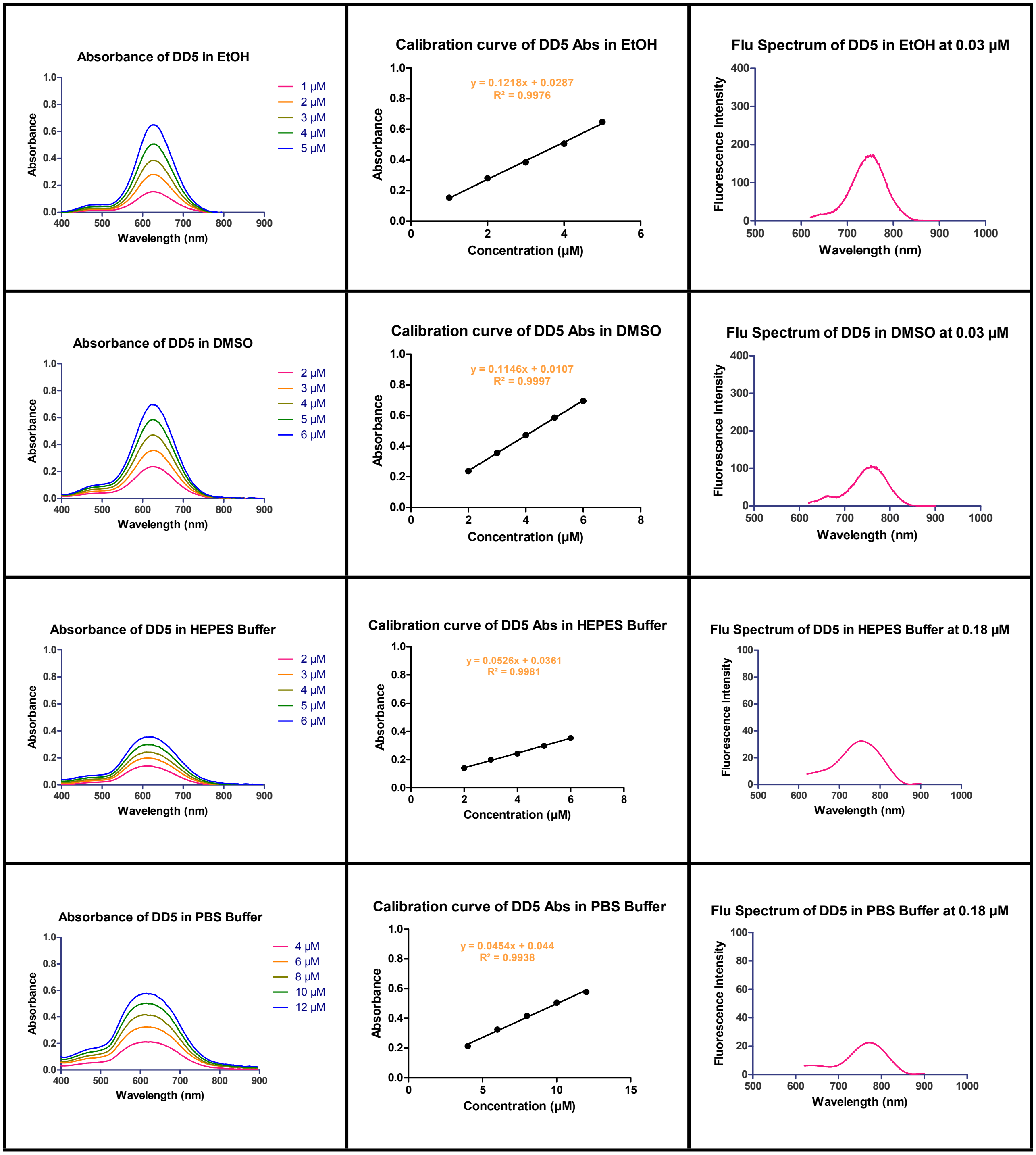

Figure S29. DD5 Absorbance curves at different concentrations, calibration curves, and fluorescence curves in different solvents

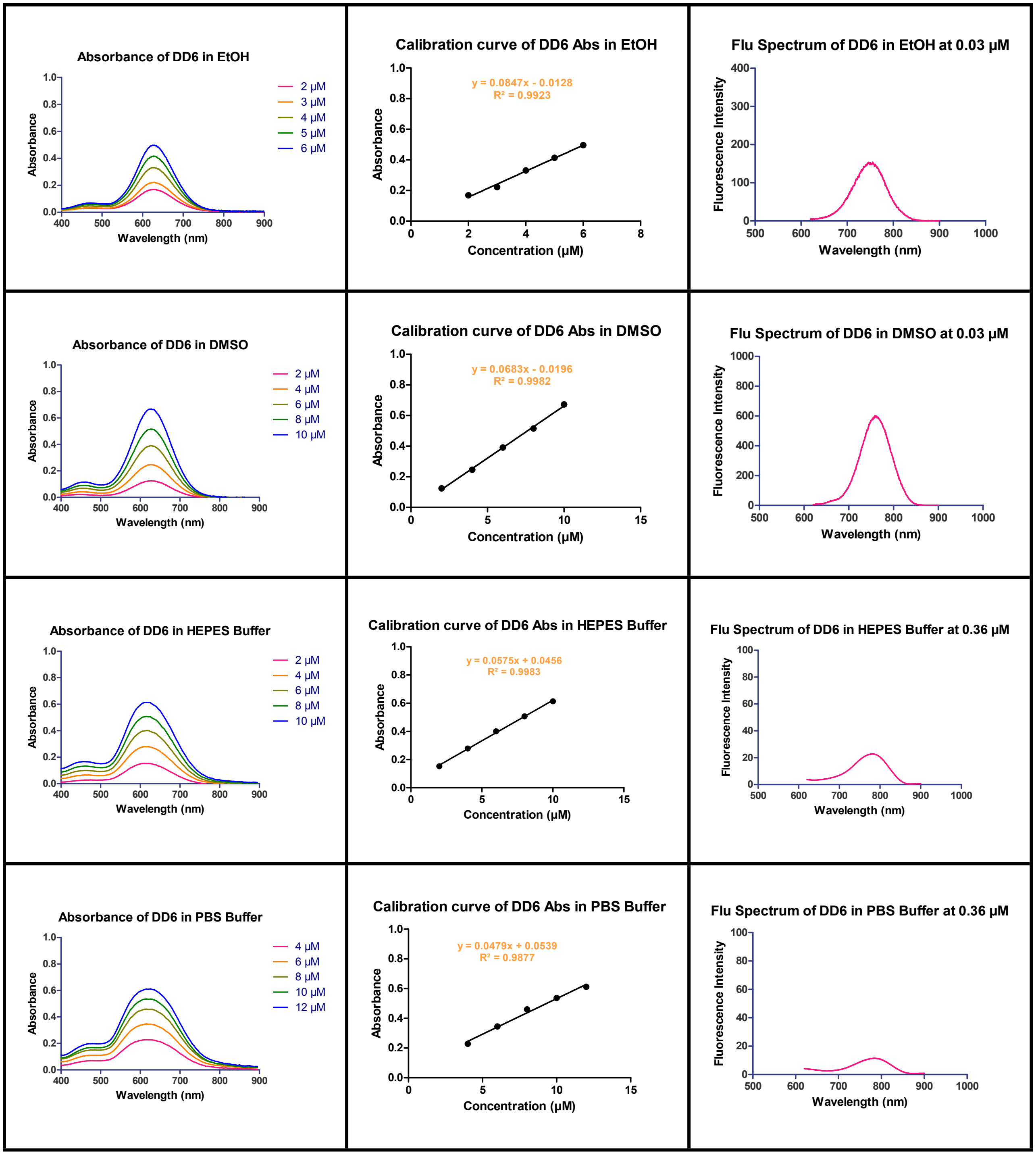

Figure S30. DD6 Absorbance curves at different concentrations, calibration curves, and fluorescence curves in different solvents

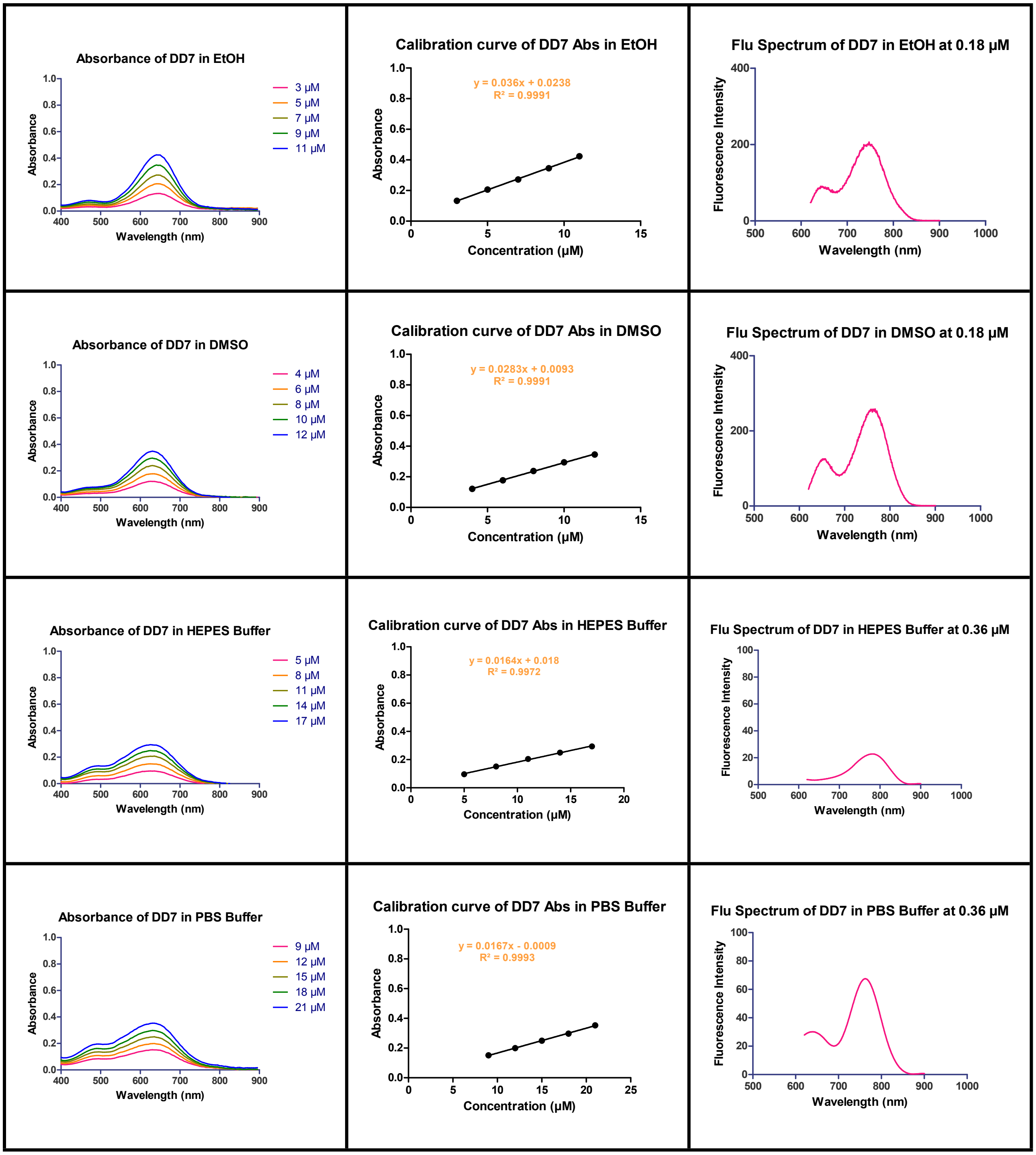

Figure S31. DD7 Absorbance curves at different concentrations, calibration curves, and fluorescence curves in different solvents

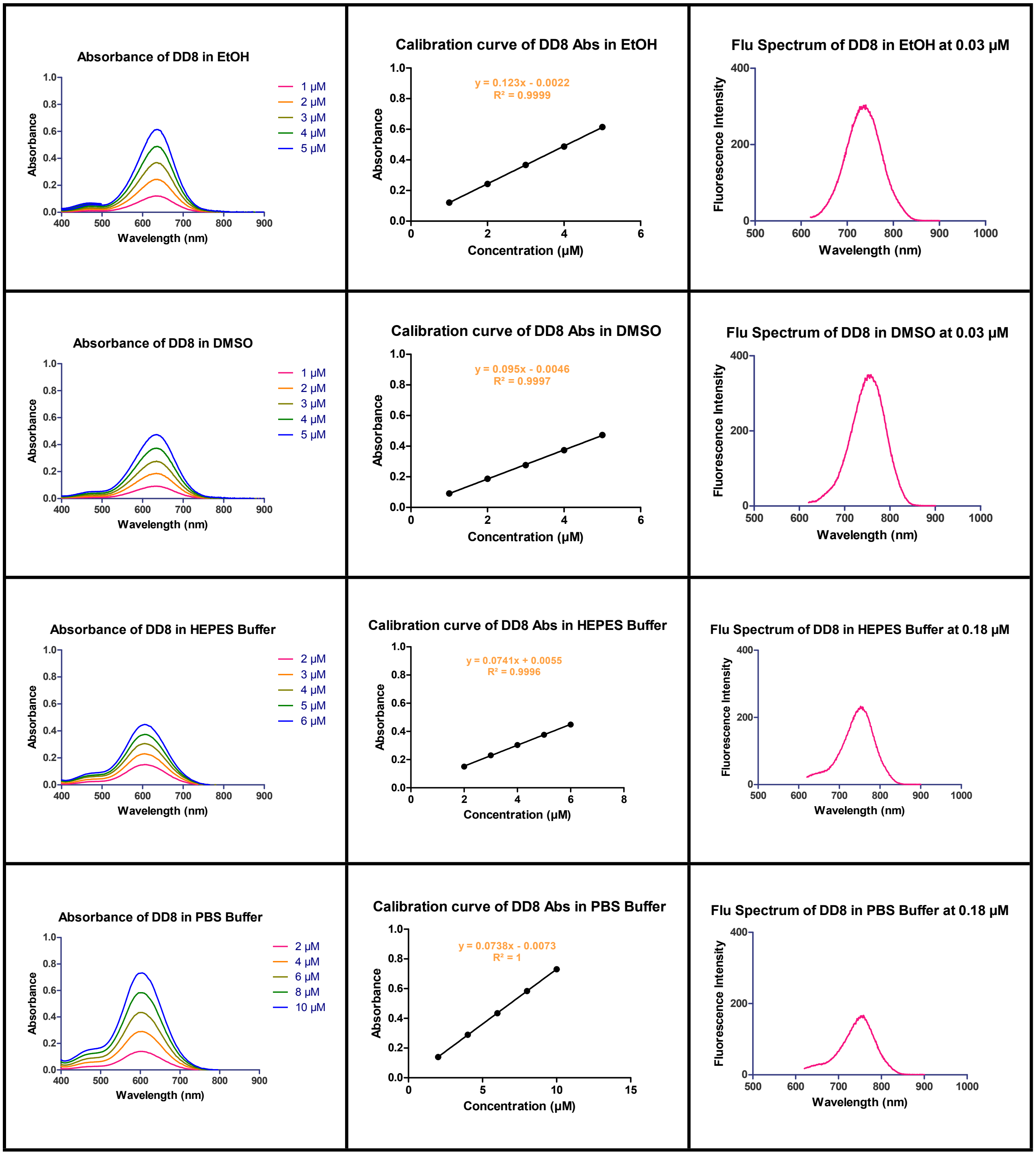

Figure S32. DD8 Absorbance curves at different concentrations, calibration curves, and fluorescence curves in different solvents

7. Photothermal stability Studies

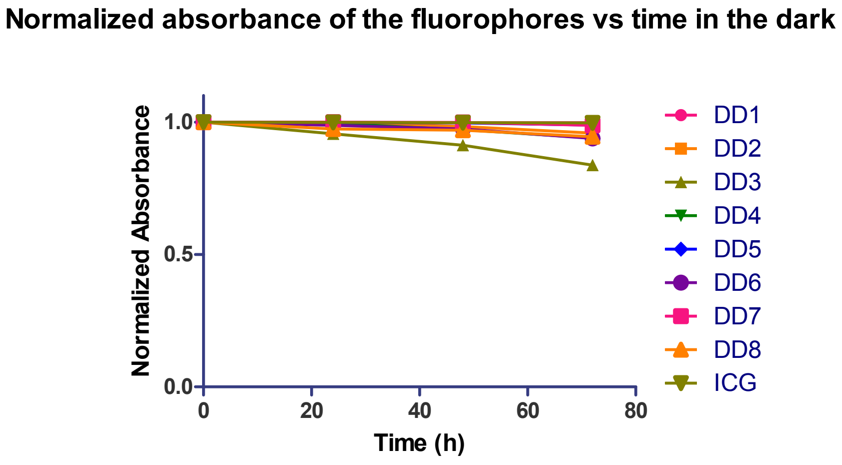

Figure S33. Photothermal stability of dimeric dyes **DD1-8**; Normalized absorbance of the synthesized dimeric dyes over time in dark conditions

8. Metal Sensing Studies

8.1. Effect of increasing concentrations of metal ions on DD1 Absorbance

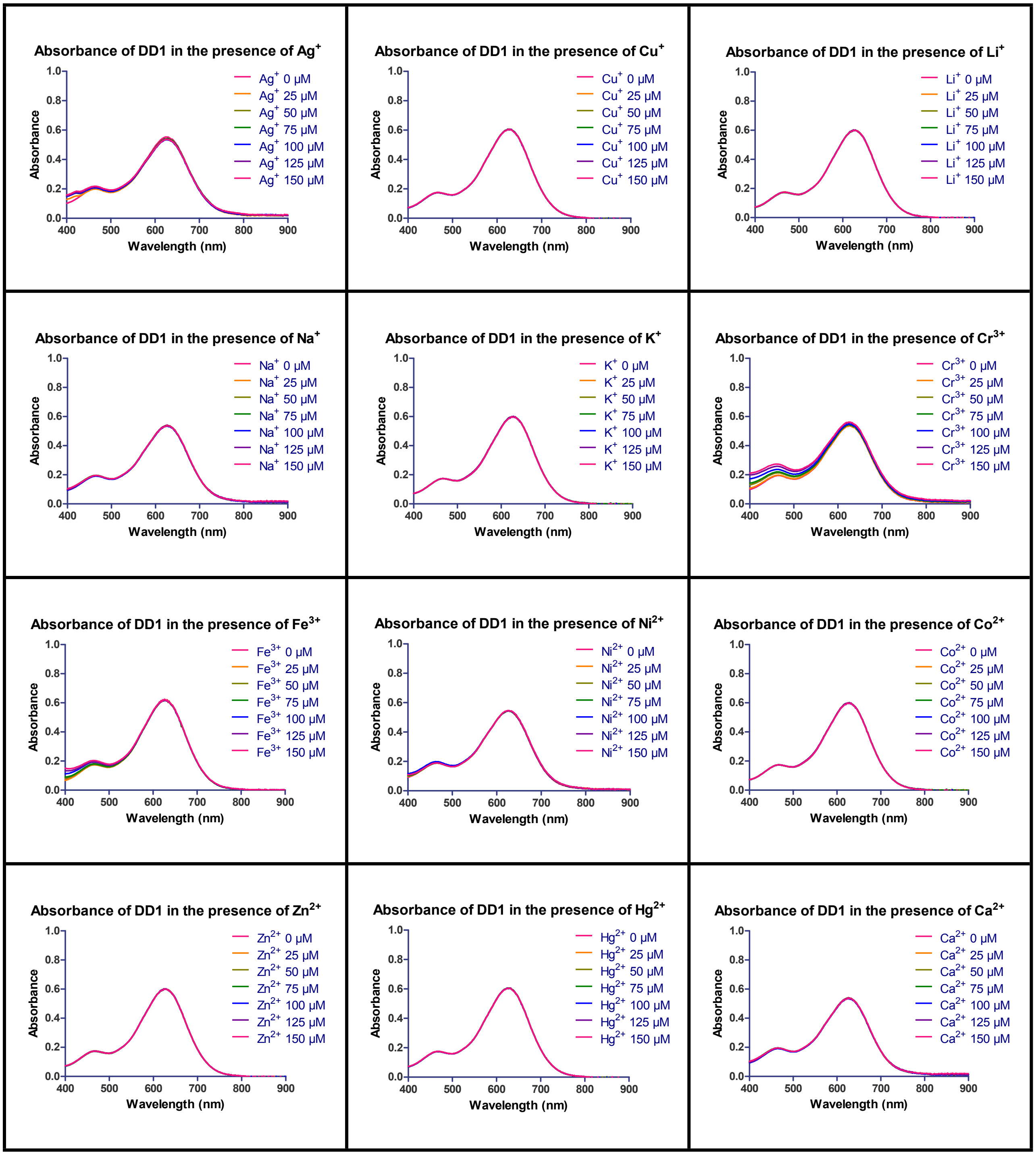

Figure S34. DD1 Absorbance spectra in the presence of increasing concentrations of different metal ions (Absorbance spectrum in the presence of Cu^{2+} is shown in the main text)

8.2. Effect of increasing concentrations of metal ions on DD1 Fluorescence

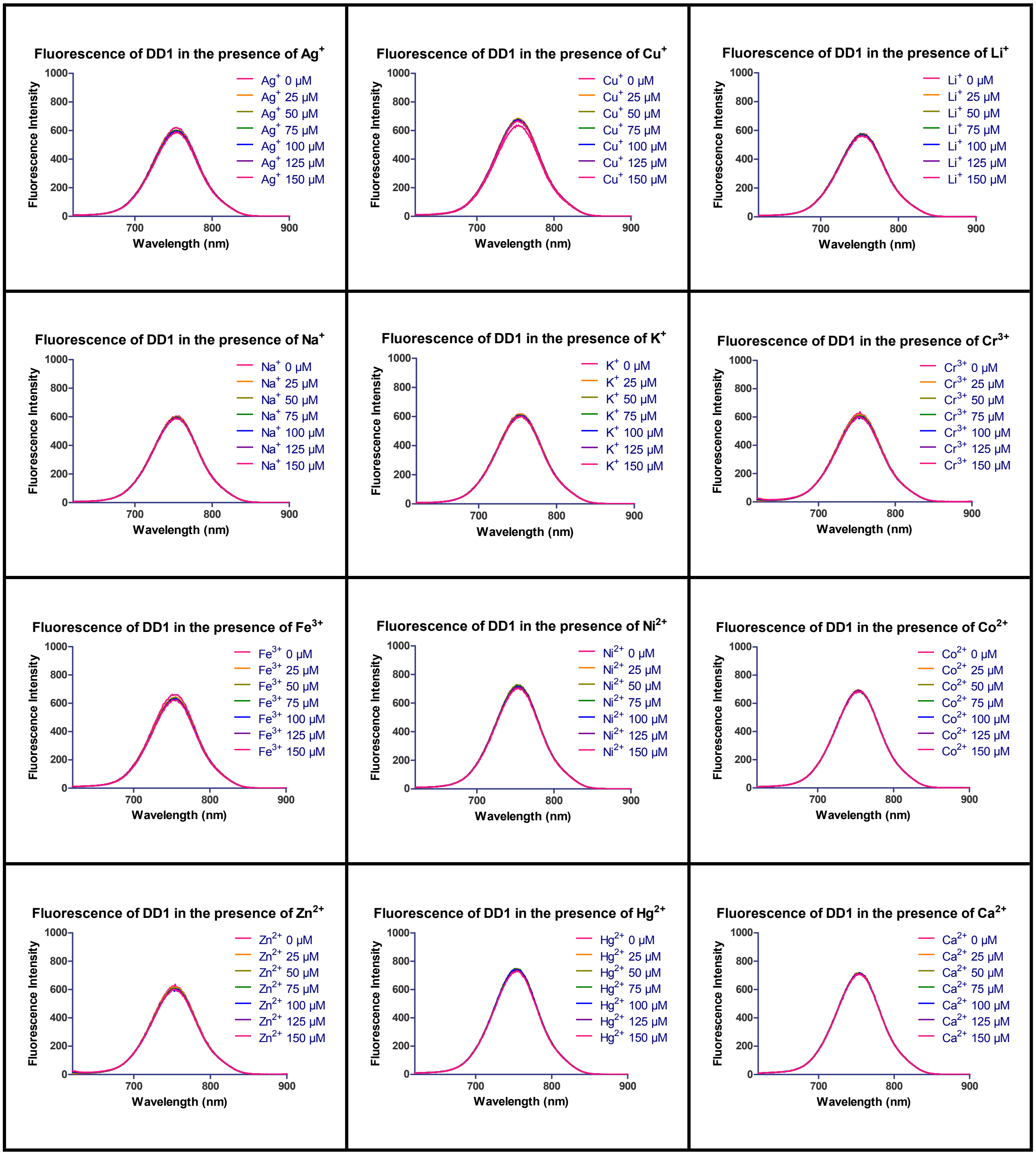

Figure S35. DD1 Fluorescence spectra in the presence of increasing concentrations of different metal ions

8.3. Effect of Cu^{2+} and other metal ions on the fluorescence intensity of DD1

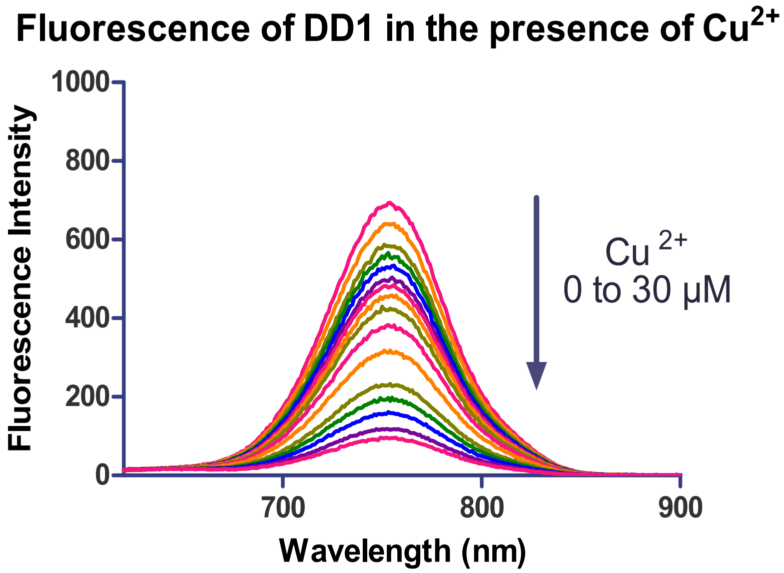

Figure S36. Effect of Cu^{2+}on the fluorescence intensity of **DD1** in 50 mM HEPES buffer

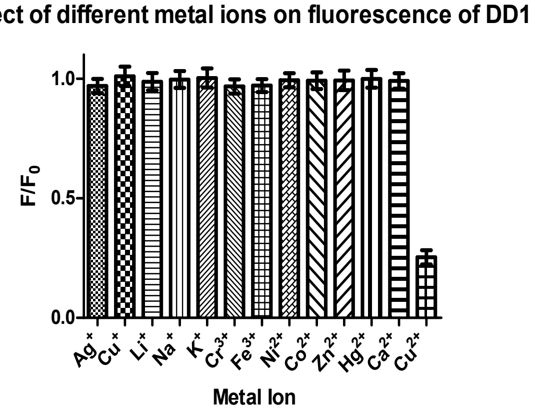

Figure S37. Effect of different metal ions on the fluorescence intensity of **DD1** in 50 mM HEPES buffer

8.4. Change in DD1 absorbance with increasing concentrations of copper (II) ions

The change in absorbance and fluorescence intensity of DD1 were plotted against the corresponding concentration of copper (II) ions to show the effect of increasing the concentration of copper (II) ions on the absorbance and fluorescence intensity respectively. This was used to calculate the limit of detection and the limit of quantitation for copper (II) ions by the fluorophore DD1. The best fitting line corresponding to the calibration curve in each graph was drawn and the limit of detection (LOD) was calculated using the equation: LOD = 3.3σ / S where σ is the standard deviation of the response or the standard error of the calibration curve and S is the slope of the calibration curve. The Limit of quantitation (LOQ) was calculated using the equation: LOQ = 10σ / S.

From the absorbance calibration curve, the LOD was calculated to be 2.5 µM, and the LOQ was calculated to be 7.6 µM, and from the fluorescence calibration curve, the LOD was 2.2 µM, and the LOQ was 6.7 µM, which is comparable to the values from the absorbance curve.

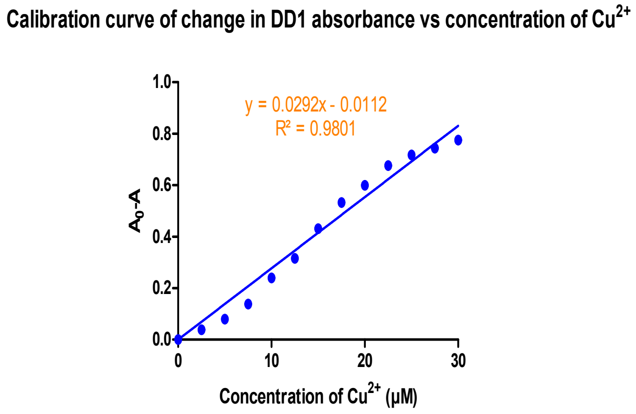

FigureS38. Calibration curve of change in DD1 absorbance with increasing concentrations of Cu^{2+} ions

8.5. Change in DD1 fl intensity with increasing concentrations of copper (II) ions

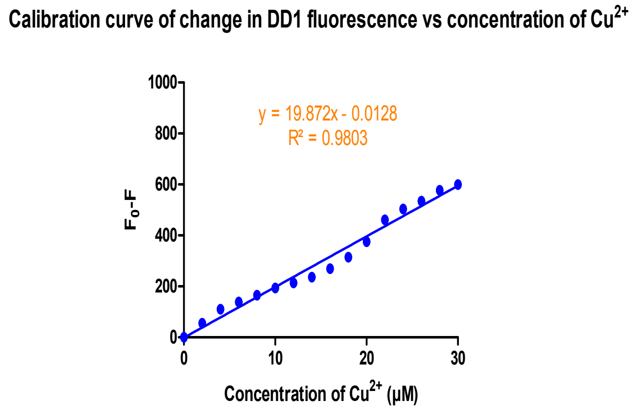

FigureS39. Calibration curve of change in DD1 fluorescence intensity with increasing concentrations of Cu^{2+} ions

8.6. Effect of increasing concentrations of metal ions on DD2 Absorbance

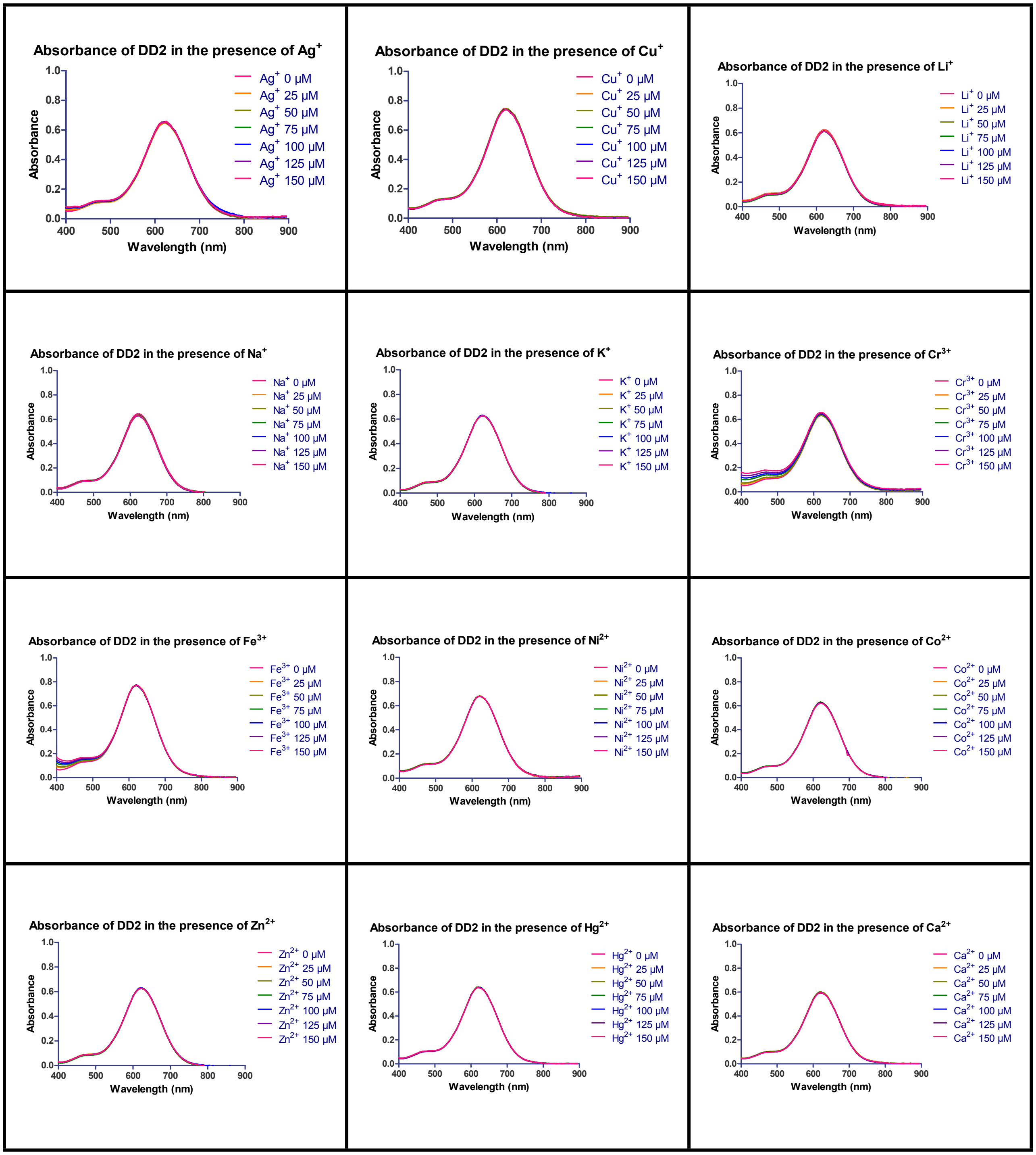

Figure S40. DD2 Absorbance spectra in the presence of increasing concentrations of different metal ions

8.7. Effect of increasing concentrations of metal ions on DD2 Fluorescence

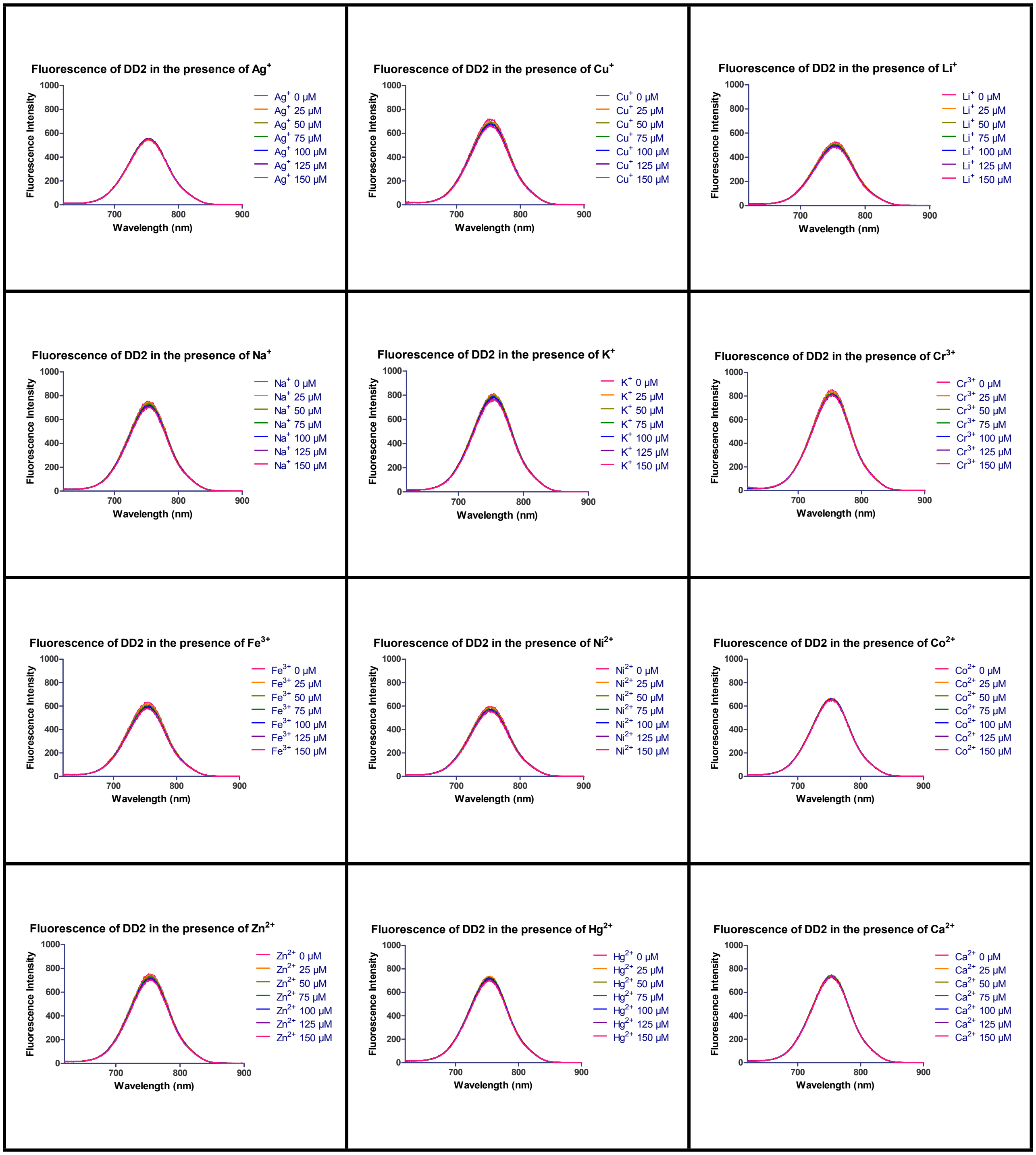

Figure S41. DD2 Fluorescence spectra in the presence of increasing concentrations of different metal ions

8.8. Effect of Cu^{2+} and other metal ions on the absorbance of DD2

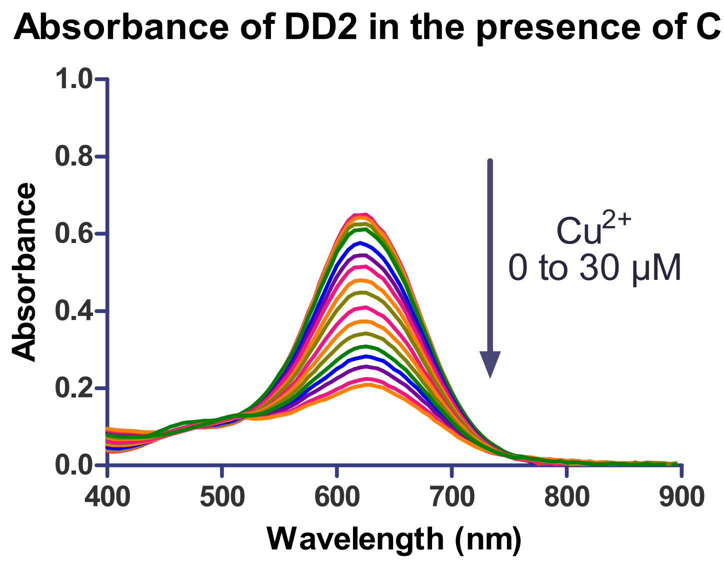

Figure S42. Effect of Cu^{2+}on the absorbance of **DD2** in 50 mM HEPES buffer

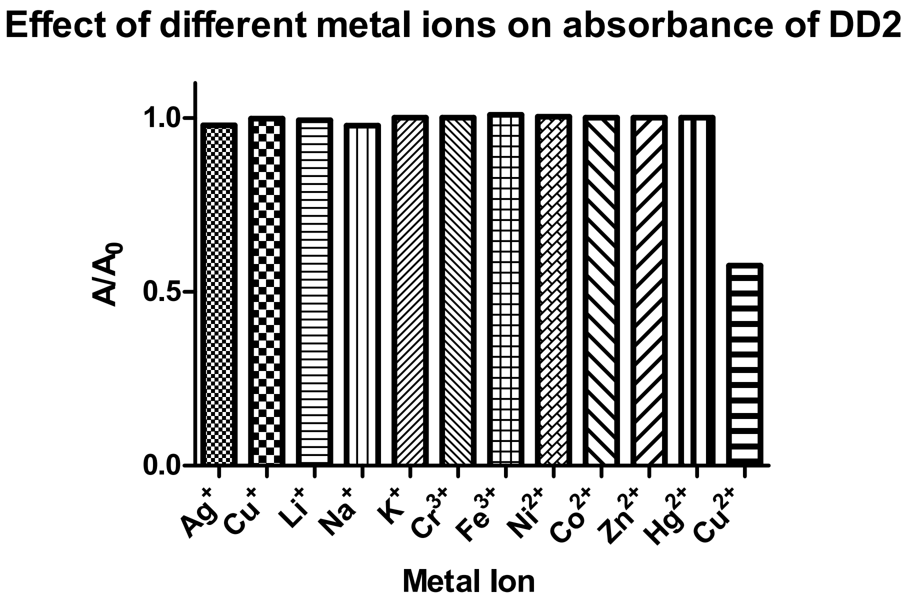

Figure S43. Effect of different metal ions on the absorbance of **DD2** in 50 mM HEPES buffer

8.9. Effect of Cu^{2+} and other metal ions on the fluorescence intensity of DD2

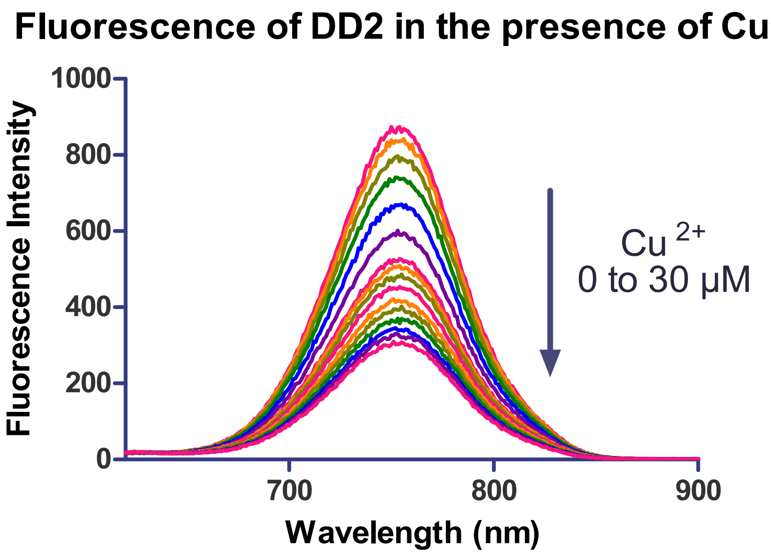

Figure S44. Effect of Cu^{2+}on the fluorescence intensity of **DD2** in 50 mM HEPES buffer

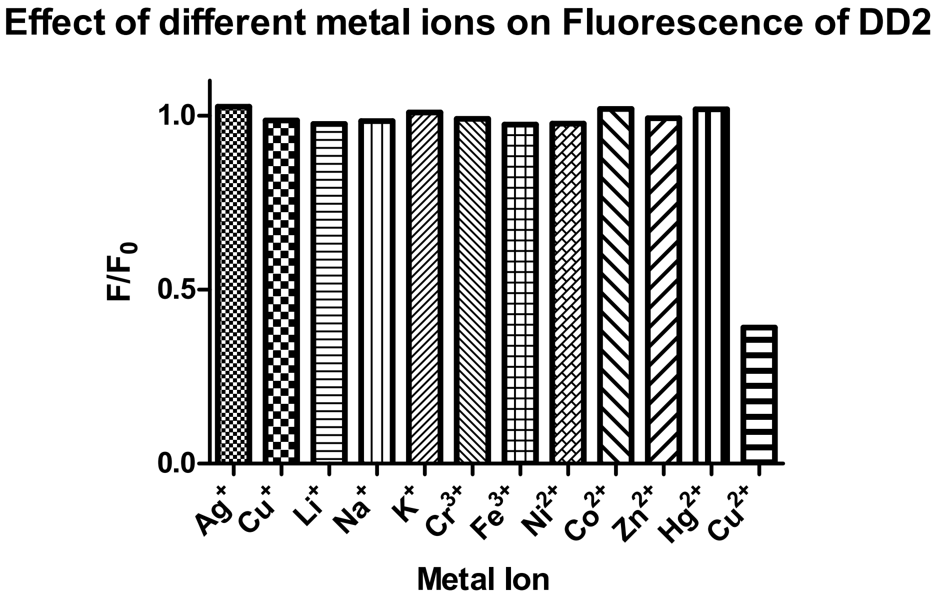

Figure S45. Effect of different metal ions on the fluorescence intensity of **DD2** in 50 mM HEPES buffer

8.10. Change in DD2 absorbance with increasing concentrations of copper (II) ions

The same method that was used for DD1 was used for the calculation of LOD and LOQ of DD2. From the absorbance calibration curve, the LOD was calculated to be 2.1 µM, and the LOQ was calculated to be 6.5 µM, and from the fluorescence calibration curve, the LOD was 3.5 µM, and the LOQ was 10.6 µM.

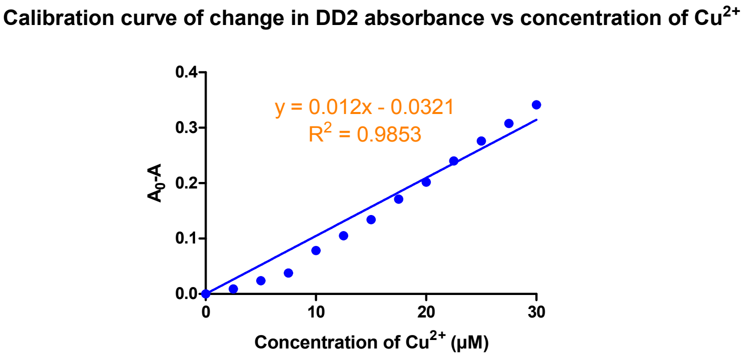

FigureS46. Calibration curve of change in **DD2** absorbance with increasing concentrations of Cu^{2+} ions

8.11. Change in DD2 fl intensity with increasing concentrations of copper (II) ions

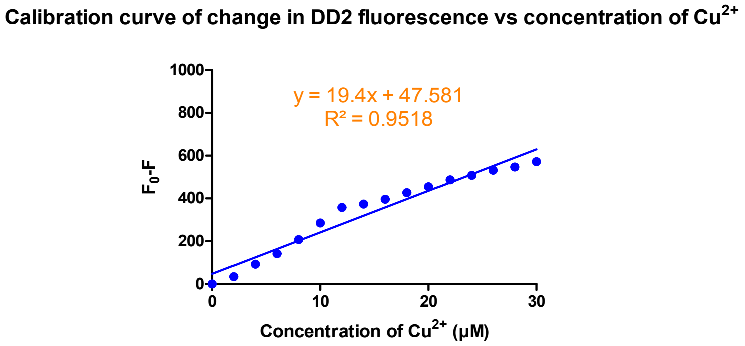

FigureS47. Calibration curve of change in **DD2** fluorescence intensity with increasing concentrations of Cu^{2+} ions

9. References

(1) Yuan, L.; Lin, W.; Zhao, S.; Gao, W.; Chen, B.; He, L.; Zhu, S. A unique approach to development of near-infrared fluorescent sensors for in vivo imaging. *Journal of the American Chemical Society* **2012**, *134* (32), 13510-13523.

(2) Shamim, M.; Dinh, J.; Yang, C.; Nomura, S.; Kashiwagi, S.; Kang, H.; Choi, H. S.; Henary, M. Synthesis, Optical Properties, and In Vivo Biodistribution Performance of Polymethine Cyanine Fluorophores. *ACS Pharmacology & Translational Science* **2023**, *6* (8), 1192-1206. DOI: 10.1021/acsptsci.3c00101.

(3) Soriano, E.; Outler, L.; Owens, E. A.; Henary, M. Synthesis of Asymmetric Monomethine Cyanine Dyes with Red-Shifted Optical Properties. *Journal of Heterocyclic Chemistry* **2015**, *52* (1), 180-184. DOI: https://doi.org/10.1002/jhet.1963.

(4) Tan, X.; Luo, S.; Wang, D.; Su, Y.; Cheng, T.; Shi, C. A NIR heptamethine dye with intrinsic cancer targeting, imaging and photosensitizing properties. *Biomaterials* **2012**, *33* (7), 2230-2239. DOI: https://doi.org/10.1016/j.biomaterials.2011.11.081.
(5) Flanagan, J. H.; Khan, S. H.; Menchen, S.; Soper, S. A.; Hammer, R. P. Functionalized Tricarbocyanine Dyes as Near-Infrared Fluorescent Probes for Biomolecules. *Bioconjugate Chemistry* **1997**, *8* (5), 751-756. DOI: 10.1021/bc970113g.
(6) Cosco, E. D.; Lim, I.; Sletten, E. M. Photophysical properties of indocyanine green in the shortwave infrared region. *ChemPhotoChem* **2021**, *5* (8), 727-734.
(7) Li, D.-H.; Smith, B. D. Deuterated Indocyanine Green (ICG) with Extended Aqueous Storage Shelf-Life: Chemical and Clinical Implications. *Chemistry – A European Journal* **2021**, *27* (58), 14535-14542. DOI: https://doi.org/10.1002/chem.202102816.
